# Ascl1 promotes tangential migration and confines migratory routes by induction of *Ephb2* in the telencephalon

**DOI:** 10.1038/srep42895

**Published:** 2017-03-09

**Authors:** Yuan-Hsuan Liu, Jin-Wu Tsai, Jia-Long Chen, Wan-Shan Yang, Pei-Ching Chang, Pei-Lin Cheng, David L. Turner, Yuchio Yanagawa, Tsu-Wei Wang, Jenn-Yah Yu

**Affiliations:** 1 Department of Life Sciences and Institute of Genome Sciences, National Yang-Ming University, Taipei 112, Taiwan; 2 Institute of Brain Science, National Yang-Ming University, Taipei 112, Taiwan; 3 Brain Research Center, National Yang-Ming University, Taipei 112, Taiwan; 4 Institute of Microbiology and Immunology, National Yang-Ming University, Taipei 112, Taiwan; 5 Institute of Molecular Biology, Academia Sinica, Taipei 115, Taiwan; 6 Molecular and Behavioral Neuroscience Institute and Department of Biological Chemistry, University of Michigan, Ann Arbor, MI 48109-2200, USA; 7 Department of Genetic and Behavioral Neuroscience, Gunma University Graduate School of Medicine, Maebashi 371-8511, Japan; 8 Department of Life Science, National Taiwan Normal University, Taipei 116, Taiwan

## Abstract

During development, cortical interneurons generated from the ventral telencephalon migrate tangentially into the dorsal telencephalon. Although Achaete-scute family bHLH transcription factor 1 (Ascl1) plays important roles in the developing telencephalon, whether Ascl1 regulates tangential migration remains unclear. Here, we found that Ascl1 promoted tangential migration along the ventricular zone/subventricular zone (VZ/SVZ) and intermediate zone (IZ) of the dorsal telencephalon. Distal-less homeobox 2 (Dlx2) acted downstream of Ascl1 in promoting tangential migration along the VZ/SVZ but not IZ. We further identified *Eph receptor B2* (*Ephb2*) as a direct target of Ascl1. Knockdown of *EphB2* disrupted the separation of the VZ/SVZ and IZ migratory routes. Ephrin-A5, a ligand of EphB2, was sufficient to repel both *Ascl1*-expressing cells *in vitro* and tangentially migrating cortical interneurons *in vivo*. Together, our results demonstrate that Ascl1 induces expression of *Dlx2* and *Ephb2* to maintain distinct tangential migratory routes in the dorsal telencephalon.

The cerebral cortex is important for executing high-order brain functions, such as sensory perception, motor control and cognition. Proper functions of the cerebral cortex require coordination between excitatory (glutamatergic) projection neurons and inhibitory (GABAergic) interneurons. Many neurological and psychological disorders, such as epilepsy, autism, schizophrenia, and Alzheimer’s disease, may be resulted from an imbalance of excitatory and inhibitory neuronal activities or the dysfunction of interneurons^1^,^2^. Interestingly, glutamatergic neurons and GABAergic interneurons are produced from different regions of the developing brain. Glutamatergic neurons are generated from neural progenitors in the dorsal telencephalon, which develops into the cerebral cortex. GABAergic cortical interneurons are remotely derived from neural progenitors in the ventral telencephalon, which develops into the striatum and globus pallidus^3^. Around 85% of cortical interneurons are generated from the medial ganglionic eminence (MGE) and the rest are generated from the caudal ganglionic eminence (CGE)^4^,^5^.

Once generated in the ventricular zone (VZ), glutamatergic neurons and GABAergic interneurons migrate toward final destinations through distinct paths. Glutamatergic neurons undergo radial migration to reach the cortical plate (CP)^6^. Interneurons take a long tangentially migratory path from the ventral telencephalon toward the striatum or the cerebral cortex^3^. When cortical interneurons reach the dorsal telencephalon, they continue migrating tangentially through three migratory routes: the marginal zone (MZ), intermediate zone (IZ) or ventricular/subventricular zone (VZ/SVZ) of the dorsal telencephalon^7^,^8^. Upon arrival at destined region, interneurons switch to migrate radially until they reach specific layers of the cerebral cortex, where they integrate into local neuronal circuits. Since cortical interneurons go through such a complicated migratory path, it is important to study the underlying regulatory mechanism.

Several transcription factors control cell fate specification, differentiation and migration in the telencephalon. Paired Box 6 (Pax6) and Neurogenin 1 and 2 (Neurog1/2) are expressed in the dorsal telencephalon and regulate cell fate specification and differentiation of glutamatergic neurons^9^,^10^. In addition, Neurog2 and Ascl1 induce expression of small GTPase genes to modulate actin dynamics therefore controlling radial migration of glutamatergic neurons^11^,^12^. In the ventral telencephalon, Dlx1 and 2, NK2 homeobox 1 (Nkx2.1), and Ascl1 specify and promote differentiation of GABAergic interneurons^13^. In addition, Dlx1/2 and Nkx2.1 orchestrate regulators for cytoskeleton dynamics, mitogenic cues and chemotactic molecules to control tangential migration^14^,^15^. While Ascl1 regulates radial migration of glutamatergic neurons in the dorsal telencephalon^11^,^16^, *Ascl1* is also abundantly expressed in the ventral telencephalon and critical for specification and differentiation of neurons generated from the ventral telencephalon^10^,^17^. However, whether Ascl1 induces genes involved in regulating tangential migration remains unknown.

Previous studies have suggested that Ascl1 might play roles in tangential migration. Nearly 60% of the *Ascl1*-expressing neurons migrate tangentially along the VZ/SVZ and IZ routes in the dorsal telencephalon^18^. Mutation of *Ascl1* leads to reduction of cortical interneurons in mice^19^, suggesting that *Ascl1* is required for specification and possibly tangential migration of cortical interneurons. Furthermore, Ascl1 induces the expression of *Dlx1*/*2* in the ventral telencephalon^17^,^20^. Since Dlx1/2 regulate the expression of genes involved in tangential migration^15^,^21^,^22^, it is possible that Ascl1 acts upstream of Dlx1/2 to regulate tangential migration of cortical interneurons.

Here, we used *in utero* electroporation to investigate the role of Ascl1 in tangential migration. We found that Ascl1 promoted tangential migration through both VZ/SVZ and IZ routes in the dorsal telencephalon, and Dlx2 acted downstream of Ascl1 to promote tangential migration through the VZ/SVZ route. Furthermore, Ascl1 induced *Ephb2*, which mediated the separation of the VZ/SVZ and IZ migratory routes. Ephrin-A5, a binding partner of EphB2, had a repulsive effect on *Ascl1*-expressing neurons *in vitro* and cortical interneurons *in vivo*. Together, our work provides evidence that Ascl1 induces *Ephb2* and the interaction between EphB2 and Ephrin-A5 is crucial for restricting interneurons to migrate on distinct routes in the dorsal telencephalon.

## Results

### *Ascl1* and *Dlx2* regulate the migratory behavior in the ventral telencephalon

**Figure 1:**
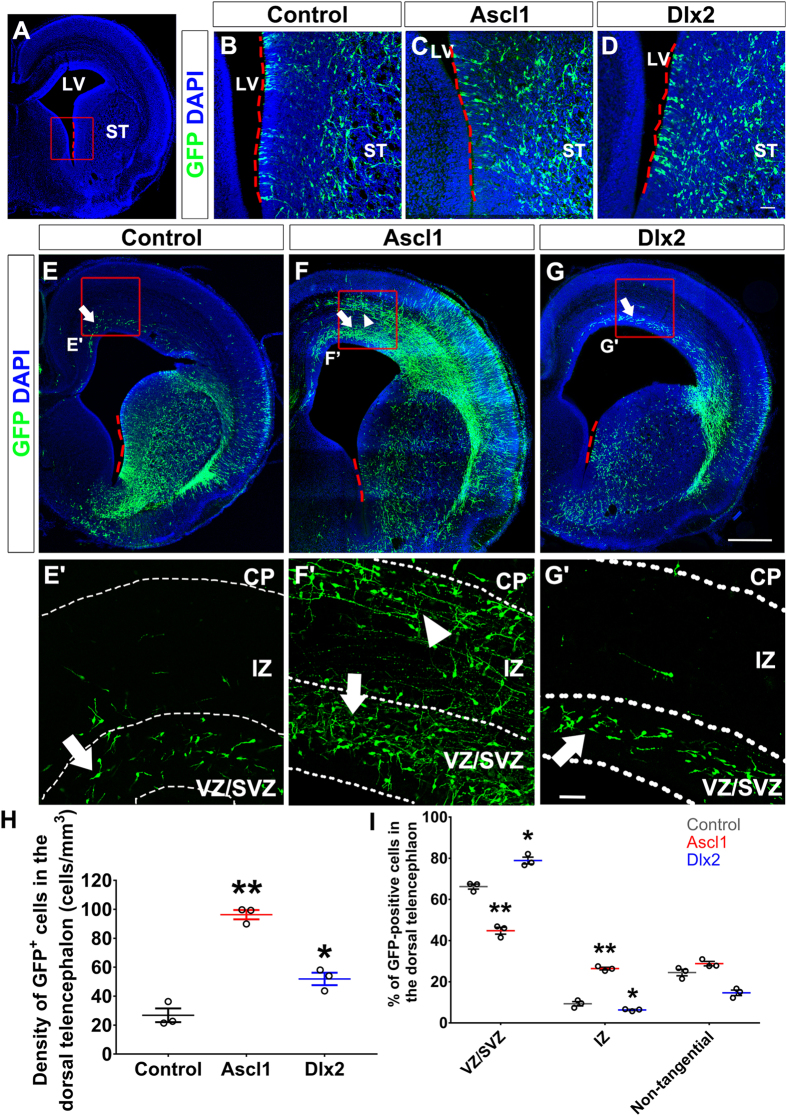
Overexpression of *Ascl1* and *Dlx2* in the ventral telencephalon promotes tangential migration. Four days after electroporation, brains of E19.5 rats were dissected and sectioned in the coronal plane. Electroporated cells were labeled with anti-GFP in green; nuclear DNA was stained with DAPI in blue. Red dashed lines indicate the electroporation region in (**A**–**G**). (**A**) A confocal image of brain section; red dashed lines mark the electroporated region and a red square indicate the area for (**B**–**D**). ST: striatum, LV: lateral ventricle. (**B**–**D**) GFP-positive cells were distributed in the ST adjacent to the LV. (**E**) In the control group, some GFP-positive cells were distributed in the VZ/SVZ (white arrows) of the dorsal telencephalon, while most remained in the ventral telencephalon. (**F**) In Ascl1 group, GFP-positive cells were distributed in both the VZ/SVZ (white arrows) and IZ (white arrowheads) of the dorsal telencephalon. (**G**) In Dlx2 group, many GFP-positive cells were distributed in the VZ/SVZ (white arrow). Red squares indicate the zoom-in areas for 1E’ to G’. Length of the scale bar is 40 μm in (**B**–**D**) and (**E’–G’**), 250 μm in (**E**–**G**). (**H**) Quantification of GFP-positive cell density in the dorsal telencephalon. (**I**) Quantification of GFP-positive cells in the VZ/SVZ, IZ, or non-tangentially migrating cells in the dorsal telencephalon. Data were presented as mean ± standard error of the mean (SEM) with all data points and analyzed by Student’s t-test, n = 3. *p < 0.05; **p < 0.01 compared to the control group.

Although knockout of *Ascl1* or *Dlx1*/*2* reduces the number of interneurons reaching the dorsal telencephalon in mice^19^,^23^, whether Ascl1 or Dlx2 promotes tangential migration has never been tested. Thus, we transfected *Ascl1* or *Dlx2* together with *GFP* expression constructs into the ventral telencephalon of rats at embryonic day 15.5 (E15.5) by using *in utero* electroporation (Fig. 1A). The parental vector US2 was used as a control. Four days after electroporation, many GFP-positive cells were observed near the ventricle of the striatum (Fig. 1B–D), demonstrating that these constructs were delivered into the ventral telencephalon successfully. In the control group, some GFP-positive cells were present in the VZ/SVZ of the dorsal telencephalon with leading processes in parallel with the ventricle (Fig. 1E), suggesting that these neurons had migrated from the ventral to the dorsal telencephalon and were migrating along the VZ/SVZ route. Overexpression of *Ascl1* or *Dlx2* dramatically increased the number of GFP-positive cells in the dorsal telencephalon (Fig. 1F–H; the density of GFP-positive cells in the dorsal telencephalon is 26.8 ± 4.8 cells/mm^3^ in control, 96.3 ± 3.2 cells/mm^3^ in Ascl1 group, and 51.9 ± 4.3 cells/mm^3^ in Dlx2 group, n = 3). Interestingly, overexpression of *Ascl1* increased GFP-positive cells in both the VZ/SVZ and IZ, while overexpression of *Dlx2* only increased GFP-positive cells in the VZ/SVZ of the dorsal telencephalon (Fig. 1F,G). Quantitative analysis further demonstrated that overexpression of *Ascl1* increased the percentage of GFP-positive cells in the IZ, while overexpression of *Dlx2* increased the percentage of GFP-positive cells in the VZ/SVZ of the dorsal telencephalon (Fig. 1I). Microtubule-associated protein 2 (MAP2) is detected in differentiating and differentiated neurons. Overexpression of *Ascl1* did not increase the percentage of GFP- and MAP2-double positive cells in the dorsal telencephalon four days after electroporation (Fig. S1), which ruled out the possibility that overexpression of *Ascl1* increased GFP-positive cells in the dorsal telencephalon through promoting neuronal differentiation. Thus, this result suggests that Ascl1 promotes tangential migration through both VZ/SVZ and IZ routes, while Dlx2 promotes tangential migration only through the VZ/SVZ route.

### *Ascl1* and *Dlx2* are sufficient to promote tangential migration in the dorsal telencephalon

**Figure 2:**
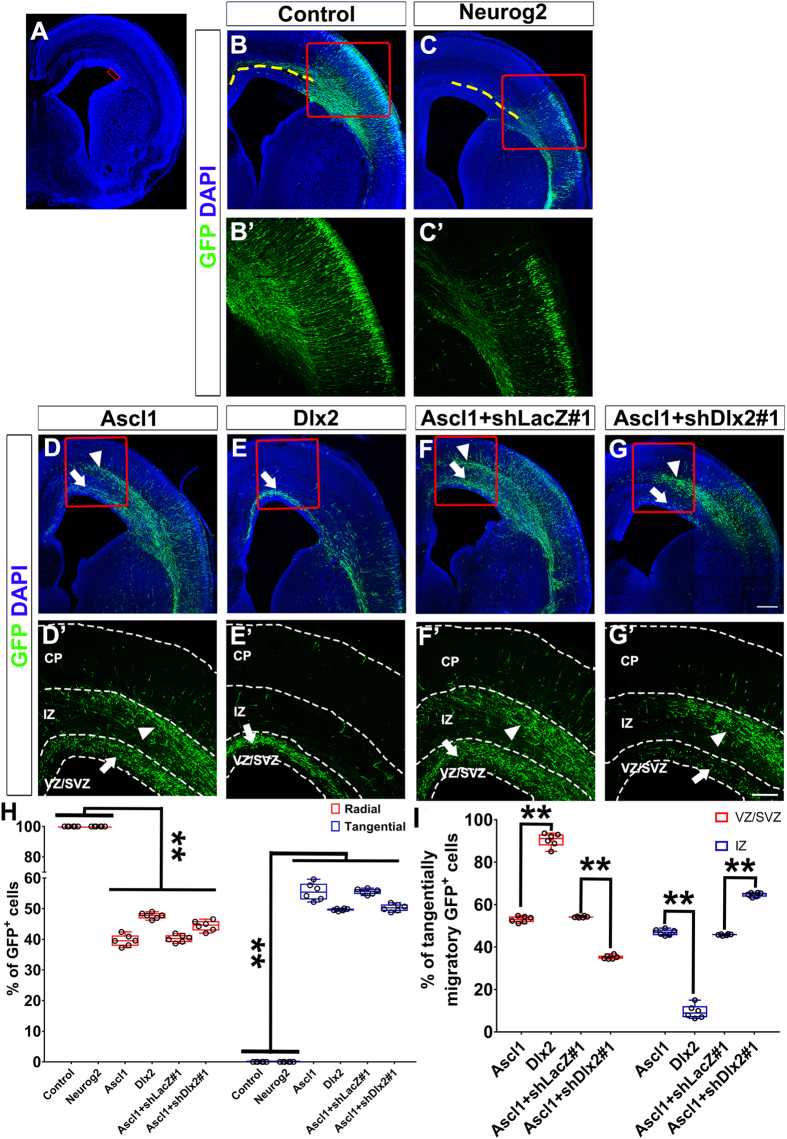
Overexpression of *Ascl1* in the dorsal telencephalon induces ectopically tangential migration. Four days after electroporation to the dorsal telencephalon, brains of E19.5 rats were dissected and sectioned in the coronal plane. Electroporated cells were labeled with anti-GFP in green; nuclear DNA was stained with DAPI in blue. (**A**) A confocal image with a red square indicating the electroporated site. (**B’–G’**) are zoomed regions of (**B–G**) indicated by red squares. (**B**) In the control group. GFP-positive cells were distributed near the electroporated area and many of them extended processes radially. GFP-positive axons toward the contralateral side were observed (yellow dashed lines). (**C**) In Neurog2 group, most GFP-positive cells were distributed in the cortical plate (CP). GFP-positive axons toward the contralateral side were observed (yellow dashed lines). (**D**) In Ascl1 group, many GFP-positive cells were distributed in the VZ/SVZ (white arrows) and IZ (white arrowheads) dorsomedially to the electroporated site. (**E**) In Dlx2 group, many GFP-positive cells were distributed in the VZ/SVZ (white arrows) dorsomedially to the electroporated site. (**F**) In Ascl1 + shLacZ#1 group, GFP-positive cells were distributed in a similar pattern as Ascl1 group in (**D**). (**G**) In Ascl1+shDlx2#1 group, many GFP-positive cells were distributed in the IZ (white arrowheads) dorsomedially to the electroporated site. Few GFP-positive cells were distributed in the VZ/SVZ (white arrows). Length of the scale bar is 120 μm in (**B**–**G**), and 100 μm in (**B’** to **G’**). (**H**) GFP-positive cells were categorized into radial, tangential, or other types according to the orientation of their leading processes. (**I**) Quantification of GFP-positive cells in the VZ/SVZ and IZ. Data are presented as box and whisker plots with all data points. The horizontal line within the box indicates the median, boundaries of the box indicate the 25th and 75th percentiles, and the whiskers indicate the maximum and minimum values of the results. Data are analyzed by using Student’s t-test, n = 6 in all groups. *p < 0.05; **p < 0.01.

**Figure 3:**
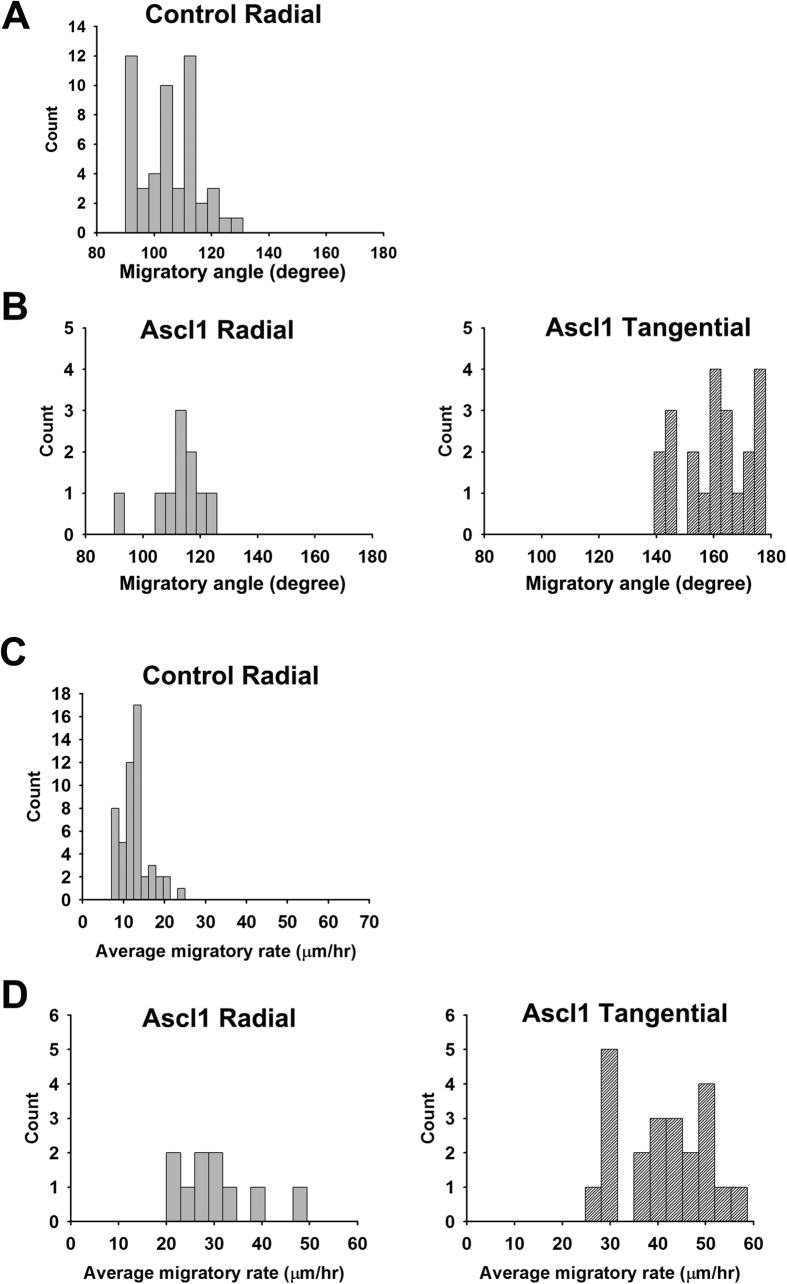
Overexpression of Ascl1 changes the migratory behavior of neurons in the dorsal telencephalon. *Ascl1* or US2 control expression construct were co-electroporated with a GFP expression plasmid. Two days after electroporation to the dorsal telencephalon, brains of E17.5 rats were dissected and sectioned in the coronal plane for slice culture and live imaging recording. The video from 5 to 11 hours after recording was used for tracking the migratory behavior. We set 0° to 180° axis in parallel to the lateral ventricle and the dorsomedial side as 180°. The line connecting the cell body location in the first frame (starting point) and the last frame (end point) of the video was used for measuring migratory angle. The average migratory rate was calculated as accumulated distance of every six-minute interval divided by recording time. 50 GFP-positive cells were counted for the control and 32 were counted for Ascl1 group. All counts were plotted into histograms according to their migratory angle or rate. (**A**) In the control group, most GFP-positive cells migrated radially in a migratory angle between 90° to 130°. (**B**) In Ascl1 group, GFP-positive cells were categorized into radially (90°–130°) and tangentially (140°–180°) migrating cells. (**C**) In the control group, GFP-positive cells migrated in the rate of 12.9 ± 3.6 μm/hour (mean ± SEM). (**D**) In Ascl1 group, radially migrating cells migrated in the rate of 30.2 ± 8.7 μm/hour and tangentially migrating cells migrated in the rate of 40.8 ± 9.3 μm/hour.

While cortical interneurons migrate tangentially into the dorsal telencephalon, glutamatergic neurons are generated in the dorsal telencephalon and migrate radially toward the CP. Thus, it would be intriguing to test whether radial migration program in neurons generated from the dorsal telencephalon can be switched to tangential migration program driven by Ascl1 or Dlx2. Expression constructs for *Ascl1 , Dlx2* or *Neurog2* was electroporated into the dorsal telencephalon together with a *GFP* expression construct (Fig. 2A). In the control group, some GFP-positive cells were distributed near the electroporated region and many GFP-positive cells reached the CP four days after electroporation (Fig. 2B). Most of these cells extended their leading processes toward the CP, suggesting that they were migrating radially (Fig. 2B’). Overexpression of *Neurog2* decreased the proportion of GFP-positive cells in the VZ/SVZ and increased the proportion of GFP-positive cells in the CP (Fig. 2C), confirming that Neurog2 promotes differentiation and radial migration in the dorsal telencephalon^12^. Similar to our findings in the ventral telencephalon, overexpression of *Ascl1* or *Dlx2* in the dorsal telencephalon increased GFP-positive cells dorsomedial to the electroporation site (Fig. 2D,E). Many GFP-positive cells in the VZ/SVZ and IZ extended their processes in parallel with the ventricle, suggesting that they were migrating tangentially (Fig. 2D’,E’). We categorized those GFP-positive cells into three groups according to the orientation of their leading processes: cells with a horizontal leading process were categorized as tangentially migrating cells; cells with a vertical leading process were radially migrating cells; cells without a distinguishable leading process were “others” (Fig. S2). Quantitative analysis showed that overexpression of *Ascl1* or *Dlx2* increased the percentage of tangentially migrating cells (Fig. 2H; the percentage was 0% in the control group, 55.3 ± 1.6% in Ascl1 group, and 49.7 ± 1.4% in Dlx2 group, n = 6). Overexpression of *Ascl1* increased the percentages of tangentially migrating GFP-positive cells in both IZ and VZ/SVZ; overexpression of *Dlx2* only increased the percentage of tangentially migrating GFP-positive cells in the VZ/SVZ (Fig. 2D,E,I; the percentage of GFP-positive cells in VZ/SVZ and IZ were 55.2 ± 1.2% and 46.8 ± 2.8% in Ascl1 group, 85.0 ± 2.0% and 15.0 ± 2.3% in Dlx2 group, n = 6). Consistently, overexpression of *Ascl1* or *Dlx2* in the dorsal telencephalon resulted in a similar distribution pattern of GFP-positive cells in mice as that in rats (Fig. S3). We further recorded live imaging of brain slices from control and Ascl1s group two days after electroporation (Fig. 3 and Supplementary videos). In the control group, most GFP-positive cells migrated radially (Fig. 3A, Video S1). In Ascl1 group, GFP-positive cells migrated either radially or tangentially (Fig. 3B, Video S2). Interestingly, the average migratory rate was dramatically increased in Ascl1 group in comparison with the control group (Fig. 3C,D), suggesting that Ascl1 promotes motility. No MAP2-positive cell was detected two days after electroporation in the IZ and VZ/SVZ of the dorsal telencephalon of control or *Ascl1* group (Fig. S4), which ruled out the possibility that overexpression of *Ascl1* changed GFP-positive cell distribution through accelerating neuronal differentiation.

To examine the possibility that Ascl1 or Dlx2 promotes tangential migration through specification of GABAergic neuronal fate, we examined markers of glutamatergic and GABAergic neurons. While most GFP-positive cells were positive for a pan-neuronal marker NeuN (96.7 ± 1.8%, n = 2) at postnatal day 13, they were not positive for the intermediate progenitor cell marker Eomesodermin (Tbr2) in the dorsal telencephalon, the glutamatergic neuronal marker vesicular Glutamate transporter 2 (vGlut2) or a GABAergic neuronal marker Gad67 at E19.5 and postnatal day 13 in rats (data not shown). Because Gad67 was difficult to detect by using immunofluorescence during embryonic stage, we used *Gad67-GFP* mice, whose GABAergic interneurons were labeled by *Gad67* endogenous promoter-driven *GFP*^24^. Control, *Ascl1*- or *Dlx2*-expression constructs were electroporated with a *DsRed*-expression construct into the dorsal telencephalon of E14.5 *Gad67-GFP* mouse embryos. Four days after electroporation, no DsRed and GFP double-positive cells were identified in the control group (Fig. S5A,D), showing that the dorsal progenitors do not differentiate into GABAergic neurons. 13.6 ± 0.8% of *Ascl1*-overexpressing cells and 36.0 ± 3.7% of *Dlx2*-overexpressing cells were *Gad67*-GFP-positive (Fig. S5B–D). In these DsRed and GFP double positive cells, 35.2 ± 4.2% of *Ascl1*-overexpressing cells and 91.0 ± 2.8% of *Dlx2*-overexpressing cells were categorized as tangentially migrating neurons (Fig. S5B,C and E). We have previously shown that over 45% of *Ascl1*- or *Dlx2*-expressing cells underwent tangential migration four days after electroporation in the dorsal telencephalon in rats (Fig. 2H). If Ascl1 and Dlx2 promote tangential migration secondary to GABAergic neuronal fate specification, we expect to see higher percentages of *Ascl1*- and *Dlx2*-overexpressing cells adapt GABAergic fate and positive for *Gad67*-GFP. Thus, our data suggest that Ascl1 and Dlx2 might promote tangential migration in parallel with re-specification of GABAergic neuronal fate.

### Ascl1 promotes tangential migration through Dlx2-dependent and Dlx2-independent manners

It has been shown that *Dlx2* is a direct target gene of Ascl1^20^. Since both Ascl1 and Dlx2 promote tangential migration, it is possible that Ascl1 promotes tangential migration by inducing *Dlx2*. To test it, expression constructs for *Ascl1* and shRNAs targeting *Dlx2* (shDlx2#1 and #2) were co-electroporated into the dorsal telencephalon. shLacZ#1 and #2 targeting *LacZ* were used as controls (Fig. S6). Transfection of shDlx2#1 or #2 efficiently knocked down *Dlx2* in P19 cells, a mouse teratocarcinoma cell line (Fig. S6B). The distribution of GFP-positive cells in the dorsal telencephalon expressing *Ascl1* and shLacZ#1 or #2 was similar to that of expressing *Ascl1* alone (Figs 2D,F, S7B). Overexpression of *Ascl1* and knockdown of *Dlx2* did not reduce the percentage of tangentially migrating cells (Fig. 2F–H; the percentage was 55.7 ± 1.3% in Ascl1+shLacZ#1 group, 50.5 ± 1.6% in Ascl1+shDlx2#1 group, n = 6). Interestingly, the percentage of GFP-positive cells in the VZ/SVZ was reduced (Fig. 2F,G,I; the percentage of Ascl1 + shLacZ#1 group was 53.9 ± 0.9%, Ascl1+shDlx2#1 group was 36.8 ± 0.9%, n = 6), while the percentage of GFP-positive cells in the IZ was increased (Fig. 2F,G,I; the percentage of Ascl1 + shLacZ#1 group was 46.3 ± 0.4%, Ascl1+shDlx2#1 group was 63.2 ± 1.1%, n = 6). A similar distribution of GFP-positive cells was observed when we knocked down Dlx2 with shDlx2#2 (Fig. S7C). This result suggests that Dlx2 acts downstream of Ascl1 in promoting tangential migration through the VZ/SVZ route. In addition, Ascl1 promotes tangential migration through the IZ route independently of Dlx2.

### EphB2 acts downstream of Ascl1 in regulating tangential migration

**Figure 4:**
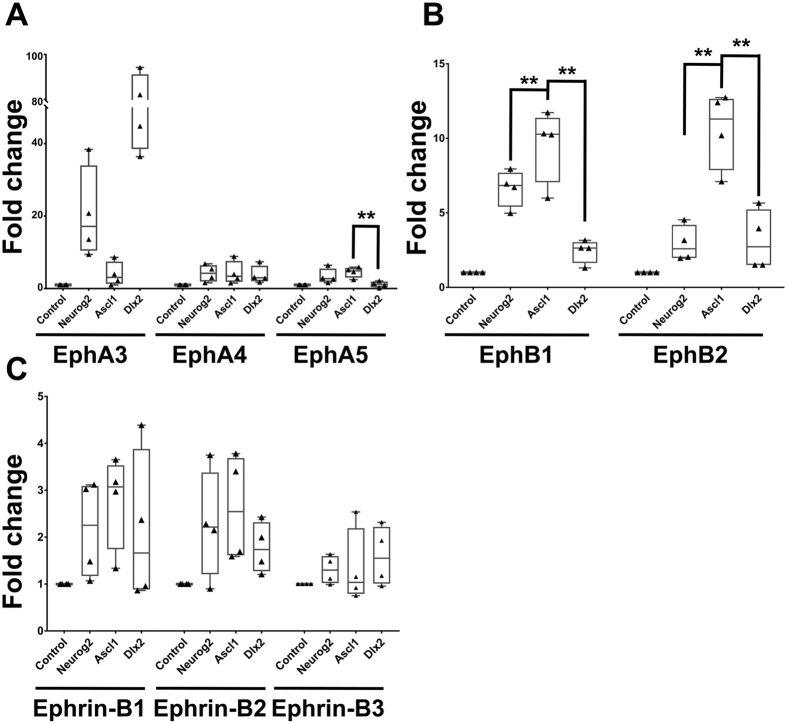
Identification of Ascl1 target genes of the Eph and Ephrin (Efn) family. P19 cells were transfected with *Neurog2 , Ascl1*, or *Dlx2* expression constructs and total RNA was extracted two days after transfection. The expression of *Eph*s (**A**,**B**) and *Efn*s (**C**) were normalized to the expression of *TATA-box binding protein* (*Tbp*). *Ephb1* and *Ephb2* were expressed at higher levels in *Ascl1-*expressing cells than those in *Neurog2* and *Dlx2*-expressing cells. Data are presented as box and whisker plots with all data points. Data are analyzed by using Student’s t-test, n = 4. *p < 0.05; **p < 0.01.

Although Rnd3 is shown to act downstream of Ascl1 to promote radial migration of glutamatergic neurons^11^, direct targets of Ascl1 involved in tangential migration of cortical interneurons have never been identified. Since overexpression of *Ascl1* led to a distinct cell distribution in the IZ and VZ/SVZ (Figs 1 and 2), it is possible that *Ascl1*-expressing cells sense repulsive cues and avoid specific areas of the dorsal telencephalon. Eph and Ephrin families are repulsive cues for axons and migrating neurons^25^,^26^. Therefore, we examined whether Ascl1 induced expression of *Ephs* or *Ephrins* (*Efn*s). P19 cells differentiate into neurons upon overexpression of *Ascl1* or *Neurog2*^27^,^28^, so we used P19 cells as a model to identify putative target genes of Ascl1. Expression constructs for *Neurog2 , Ascl1*, or *Dlx2* were transfected into P19 cells and total RNA was extracted two days after transfection. By using quantitative RT-PCR (qRT-PCR), we examined the expression level of *Eph*s and *Efn*s that have been detected in the developing telencephalon^29^. Because Neurog2 promotes differentiation of glutamatergic neurons^17^, genes induced by both Neurog2 and Ascl1 were excluded. Since Ascl1 activates *Dlx2*^17^,^20^, genes induced by Dlx2 preferentially may be indirectly regulated by Ascl1. Thus, genes induced at higher levels by Ascl1 than Neurog2 and Dlx2 were selected as Ascl1 targets. *Ephb1* and *b2* mRNA were induced at higher levels by expression of *Ascl1* than that of control and expression of *Neurog2* or *Dlx2* 48 hours after transfection (Fig. 4). *Ephb1* and *b2* were induced dramatically: 9.2 ± 1.2 fold and 9.4 ± 1.1 fold relative to the control, respectively. Thus, we selected *Ephb1* and *b2* as candidate target genes and tested whether they act downstream of Ascl1 in regulating tangential migration.

**Figure 5:**
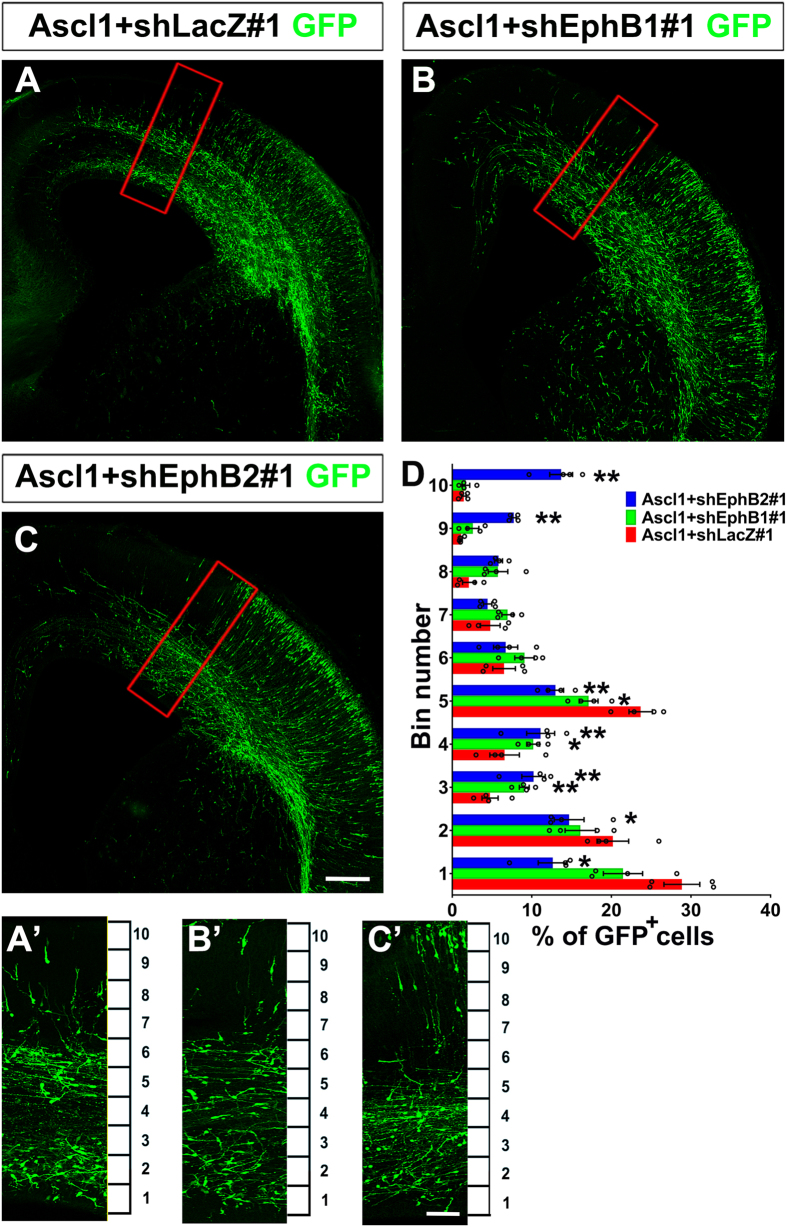
Knockdown of *Ephb1* or *Ephb2* disrupted tangential migration promoted by Ascl1. Four days after electroporation to the dorsal telencephalon, brains of E19.5 rats were dissected and sectioned in the coronal plane. Electroporated cells were labeled with anti-GFP in green. (**A’–C’**) are zoomed regions of (**A**–**C**) indicated by red squares, which were dorsomedial to the electroporated site. The cortex was divided equally into 10 bins. (**A**) In Ascl1 + shLacZ#1 group, many GFP positive cells were distributed in the VZ/SVZ (bins 1 and 2) and IZ (bin 5). (**B**,**C**) In Ascl1 + shEphB1#1 and Ascl1 + shEphB2#1 groups, GFP-positive cells were distributed in the VZ/SVZ (bins 1 and 2) and IZ (bin 5) were reduced. Length of the scale bar is 120 μm in (**A**–**C**), and 40 μm in (**A’–C’**). (**D**) Distribution of GFP-positive in the cortex. Only GFP-positive cells dorsomedial to the electroporated site were counted. Data were presented as mean ± SEM with all data points and analyzed by one-way ANOVA with Tukey’s-HSD post hoc test, n = 4. *p < 0.05; **p < 0.01 compared to Ascl1 + shLacZ#1 group.

Expression constructs for *Ascl1* and shRNAs against *Ephb1* or *b2* were electroporated into the dorsal telencephalon. *Ephb1* or *b2* mRNA expression was effectively inhibited by shRNAs against each gene (Fig. S6B). Knockdown of *Ephb1* or *b2* in *Ascl1*-overexpressing cells affected the distribution of GFP-positive cells. Importantly, the separation between the VZ/SVZ and IZ routes was disrupted (Fig. 5A–C, Fig. S7D,E). For quantification, the developing cortex was divided into 10 bins and the number of GFP-positive cells in each bin was counted. In Ascl1 + shLacZ#1 group, many GFP-positive cells were in bin 1 and 2 (the VZ/SVZ), as well as bin 5 (the IZ) (Fig. 5A’,D). Expression of *Ascl1* and knockdown of *Ephb1* slightly decreased the percentage of GFP-positive cells in bin 1, 2, and 5, but increased the percentage of cells in bin 3 and 4 comparing with Ascl1 + shLacZ#1 (Fig. 5B’,D). Expression of *Ascl1* and knockdown of *Ephb2* decreased the percentage of GFP-positive cells in bin 1, 2, and 5, but increased the percentage of cells in bin 3, 4, 9, and 10 comparing with Ascl1 + shLacZ#1 (Fig. 5C’,D). Knockdown of *Ephb1* or *b2* alone in the dorsal telencephalon did not affect distribution of electroporated cells in the CP (Fig. S8), showing that *Ephb1* and *b2* are not required for radial migration. These data suggest that EphB1 and B2 act downstream of Ascl1 in regulating the separation of the VZ/SVZ versus IZ routes during tangential migration.

**Figure 6:**
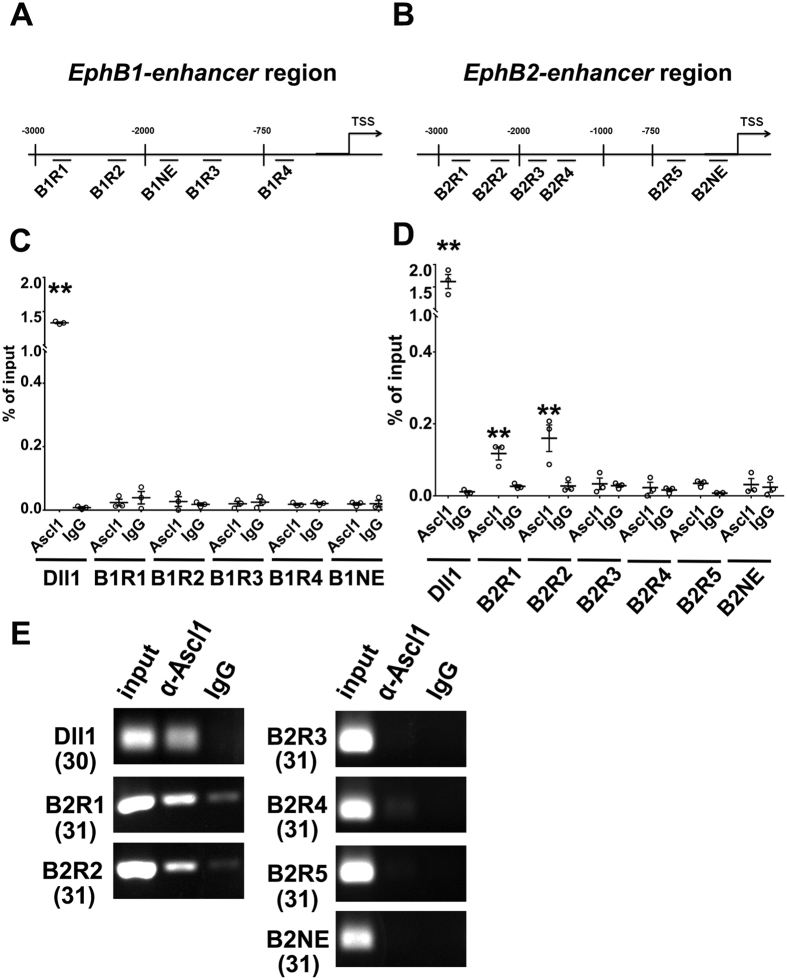
*Ephb2* is a direct target of Ascl1. (**A**,**B**) Putative E-box sequences were identified in the 3 Kb upstream of the transcription starting site (TSS) for *Ephb1* and *Ephb2*. Four fragments (B1R1 to B1R4) of *Ephb1* and five fragments (B2R1 to B2R5) of *Ephb2* containing E-box sequences were designed for ChIP. Two fragments (B1NE and B2NE) without E-box sequences were selected as negative controls. (**C**,**D**) DNA from P19 cells transfected with *Ascl1* expression vectors was extracted for ChIP. A fragment in the promoter region of *Dll1* that has been demonstrated to interact with Ascl1 was used as a positive control. After ChIP, enrichment of the DNA fragments was quantified by qPCR (**C**,**D**) and regular PCR (**E**). (**C**) The promoter region of *Dll1* was enriched by ChIP with anti-Ascl1. No fragment in *Ephb1* was enriched. (**D**) The promoter region of *Dll1*, as well as *B2R1* and *B2R2* sites of *Ephb2* were enriched by ChIP with anti-Ascl1. (**E**) Regular PCR result. The number in the parentheses indicated PCR cycle. Data were presented as mean ± SEM with all data points and analyzed by Student’s t-test, n = 3; *p < 0.05 compared to the IgG control.

We further used Chromatin immunoprecipitation (ChIP) to check whether *Ephb1* and *b2* were direct targets of Ascl1. We analyzed three kilo-bases (Kb) upstream of the transcription start site (TSS) for E-box sequences (CACCTG, CAGATG, or CAGGTG) that are potential binding sites for Ascl1^30^,^31^. Four putative E-boxes upstream of *Ephb1* (designated as B1R1 to B1R4), and five upstream of *Ephb2* (designated as B2R1 to B2R5) were identified (Fig. 6A,B). DNA was extracted from P19 cells transfected with an *Ascl1* expression construct. A DNA fragment of *Delta-like 1* (*Dll1*) with confirmed E-boxes was used as a positive control^32^. B1NE upstream of *Ephb1* and B2NE upstream of *Ephb2* without putative E-box were selected as negative controls. None of the DNA fragments upstream of *Ephb1* was precipitated by anti-Ascl1 (Fig. 6C), suggesting that Ascl1 may not induce *Ephb1* directly. Alternatively, Ascl1 may interact with an *Ephb1* enhancer outside of the 3Kb-fragment upstream of the TSS that we examined. B2R1 and B2R2 of *Ephb2* were precipitated by anti-Ascl1 (Fig. 6D,E), demonstrating that Ascl1 binds *Ephb2* directly. Neither the negative controls (B1NE and B2NE) nor B2R3, B2R4, B2R5 were precipitated by anti-Ascl1 (Fig. 6D,E). Thus, we identified *Ephb2* as a direct target of Ascl1.

We further examined whether EphB2 could be detected in tangentially migrating cortical interneurons. Overexpression of *Ascl1* in P19 cells increased the signal intensity of EphB2 detected by immunofluorescent staining (Fig. S9), which is consistent with our qPCR data (Fig. 4). Knockdown of *Ephb2* decreased the signal intensity of EphB2 (Fig. S9), demonstrating the specificity of anti-EphB2. Furthermore, in *Gad67-GFP* E18.5 mouse embryos, most GFP-positive cells in the VZ/SVZ of the dorsal telencephalon were also EphB2-positive (Fig. S10), confirming that *EphB2* is expressed in tangentially migrating cortical interneurons.

### Ephrin-A5 acts as a repulsive cue for tangentially migrating neurons

**Figure 7:**
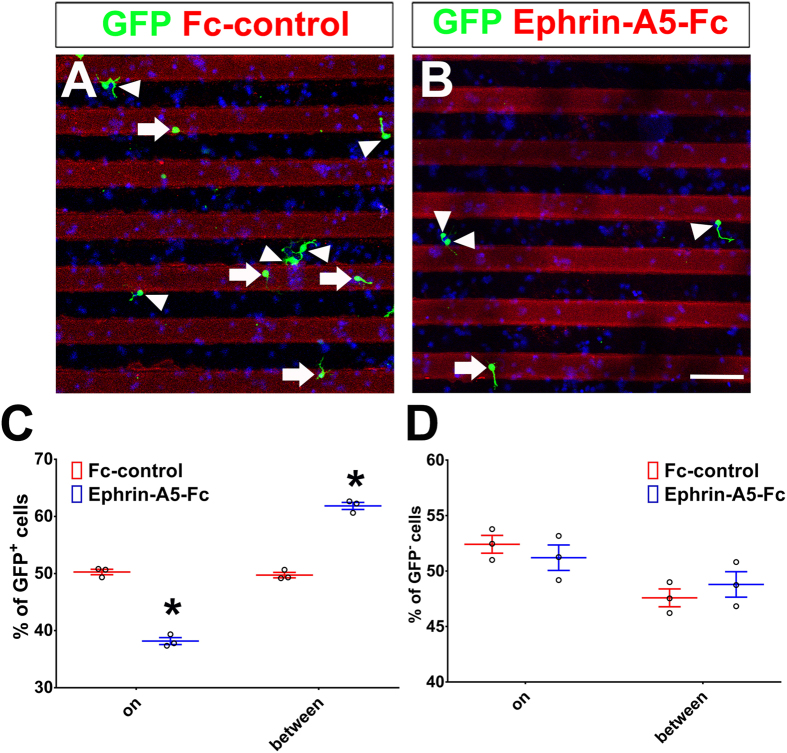
Ephrin-A5 has a repulsive effect on *Ascl1*-expressing cortical neurons. Control (US2) or *Ascl1* expression constructs were electroporated into the dorsal telencephalon of E15.5 rats. The dorsal telencephalon was dissected two days after electroporation and dissociated into individual cells. These cells were cultured on coverslips coated with Fc-control or Ephrin-A5-Fc stripes. Cells were fixed 16–18 hours after plating and cells on strips or between strips were counted. Electroporated cells were labeled with anti-GFP in green, nuclear DNA was stained with DAPI in blue. GFP-positive cells on stripes are indicated by white arrowheads; GFP-positive cells between stripes are indicated by white arrows. (**A**) On a coverslip coated with Fc-control, GFP-positive *Ascl1*-expressing cells and GFP-negative cells were distributed evenly on stripes and between stripes. (**B**) On a coverslip coated with Ephrin-A5-Fc, GFP-positive *Ascl1*-expressing cells were preferentially distributed between stripes, while GFP-negative cells were evenly distributed on stripes and between stripes. Length of the scale bar is 50 μm. (**C**,**D**) Distribution of GFP-positive and GFP-negative cells. 150 cells from each group were counted in each experiment. Data are presented as mean ± SEM with all data points and analyzed by using Chi-square test, n = 3. *p < 0.05 compared to the Fc-control.

Since our results show that *Ephb2* is a direct target of Ascl1 and is required for the separation of the VZ/SVZ and IZ routes, EphB2 interacting Ephrin(s) should be present in specific areas of the dorsal telencephalon to repel tangentially migrating cortical interneurons. Previous studies have shown that Ephrin-A5 and Ephrin-B2 potentially interact with EphB2^33^,^34^. *Ephrin-A5* (*Efna5*) is expressed in the MGE and preoptic area (POA) of the ventral telencephalon and acts as a repulsive cue for interneurons^35^. *Efna5* mRNA and Ephrin-A5 protein have been detected in the CP of the dorsal telencephalon^36^,^37^. We examined the distribution of Ephrin-A5 in the telencephalon of E19.5 rat embryo by immunofluorescence. High levels of Ephrin-A5 were detected in deep VZ, upper SVZ, deep IZ and CP, but the signal was lower in the VZ/SVZ and IZ routes for tangential migration (Fig. S11). This result suggests that Ephrine-A5 may serve as a repulsive cue for cortical interneurons. To test this hypothesis, recombinant Ephrin-A5-Fc chimera (Ephrin-A5-Fc) or control protein (Fc-control) was coated onto 50 μm-wide stripes with Alexa 549 donkey-anti-guinea pig antibody for visualizing the coated area. Expression constructs of *Ascl1* and *GFP* were co-electroporated into the dorsal telencephalon. Cells near the electroporated site were dissected two days after electroporation and cultured on the coverslips with coated stripes. 16 to 18 hours after plating, GFP-negative cells were equally distributed on stripes and between stripes of Fc-control or Ephrin-A5-Fc (Fig. 7D). While *Ascl1*-expressing GFP-positive cells were distributed equally on and between Fc-control stripes, they were preferentially distributed between Ephrin-A5-Fc stripes (Fig. 7A–C; mean ± SEM = 49.7 ± 0.8% in Fc-control group, 61.8 ± 1.1% in Ephrin-A5-Fc group, n = 3) and avoided the Ephrin-A5-Fc coated stripes (Fig. 7A–C; 50.3 ± 0.8% in Fc-control group, 38.2 ± 1.1% in Ephrin-A5-Fc group, n = 3). This result suggests that Ephrin-A5 acts as a repulsive cue for *Ascl1*-expressing cells.

**Figure 8:**
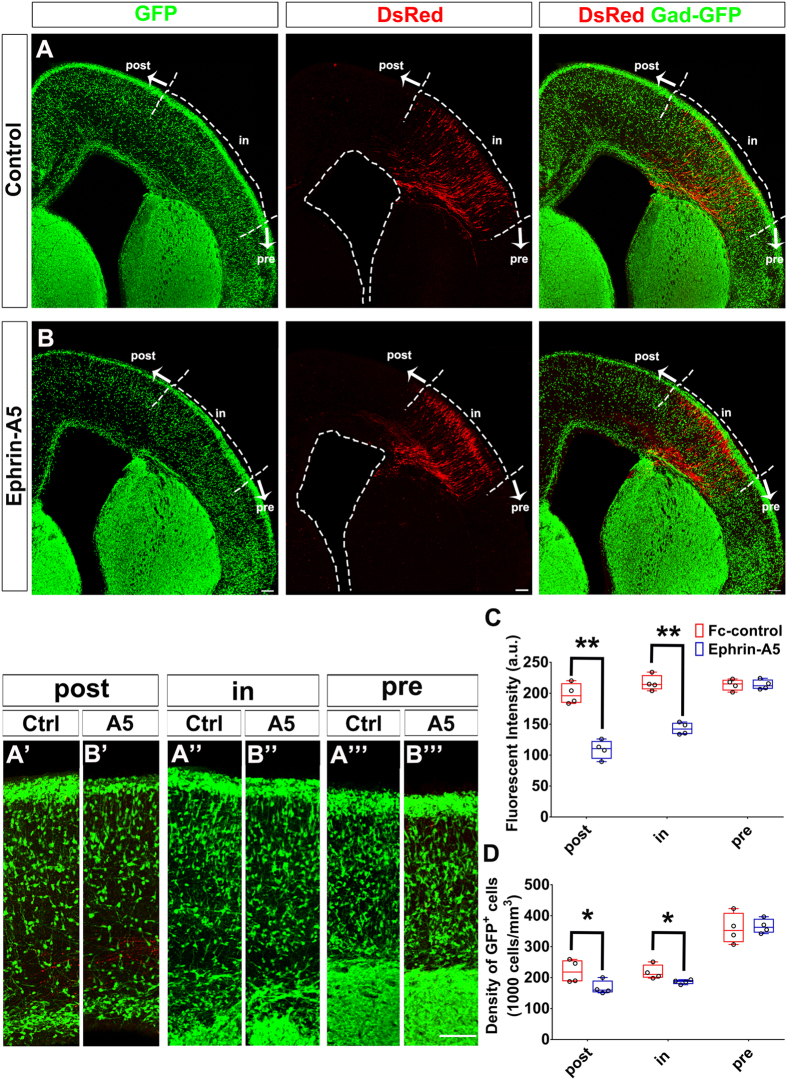
Ephrin-A5 repulses cortical interneurons. Control (US2) or *EfnA5* expression constructs were electroporated into the dorsal telencephalon together with a *DsRed* expression construct at E14.5 of *Gad67-GFP* mice. Brains were dissected four days after electroporation and sectioned coronally. (**A**,**B**) Confocal image of E18.5 mouse telencephalon. The cortex was divided into three regions: (A”,B”) the “in” region that contained DsRed-positive cells; (A”’,B”’) the “pre” region was ventrolateral to the “in” region; (A’,B’) the “post” region was dorsomedial to the “in” region. (**A**,**B**) In Ephrin-A5 group, fewer GFP-positive cells were present in “post” regions than those of the control group. Length of the scale bar is 100 μm. (**C**) Quantification of GFP fluorescent intensity in the MZ. (**D**) Quantification of GFP-positive cells in the CP, IZ and VZ/SVZ. A 200 μm wide cortical area in each region was selected for quantification. For the pre-DsRed, we selected the 200 μm wide cortical area 50 to 100 μm from the pre-/in-DsRed boundary. For the in-DsRed, we selected 50 to 100 μm from the in-/post-DsRed boundary. For the post-DsRed, we selected 50 to 100 μm from the in-/post-DsRed boundary. Data are presented as box and whisker plots and analyzed by using Student’s t-test, n = 4. *p < 0.05.

To investigate whether Ephrin-A5 repulses tangentially migrating cells *in vivo*, we examined interneuron distribution in *Gad67-GFP* mice. Expression constructs of *Efna5* and *DsRed* were co-electroporated into the dorsal telencephalon at E14.5. Based on our hypothesis, these *Efna5*-expressing DsRed-positive cells should repel migrating GFP-positive cortical interneurons. In comparison with the control group, fewer GFP-positive cortical interneurons were observed dorsomedial to the region containing DsRed-positive *Efna5*-expressing cells (Fig. 8A,B). We quantified the average fluorescent intensity and the density of GFP-positive cells in the pre-, in-, or post-DsRed region (Fig. 8A,B). In comparison with the control group, the average fluorescent intensity of the mental zone (MZ) was reduced in the post-DsRed (Fig. 8C; control: 198.9 ± 8.4, Ephrin-A5: 109.1 ± 7.6; n = 4) and in-DsRed regions (Fig. 8C; control: 216.6 ± 6.3, Ephrin-A5: 142.8 ± 4.9; n = 4) when *Efna5* was over-expressed. The density of GFP-positive cells of the CP, IZ, SVZ/VZ was also reduced in the post-DsRed region when *Efna5* was over-expressed (Fig. 8C; post-DsRed, control: 2.2 × 10^5^ ± 1.9 × 10^4 ^cells/mm^3^, Ephrin-A5: 1.7 × 10^5^ ± 1.1 × 10^4 ^cells/mm^3^; n = 4). This result indicates that Ephrin-A5 repels tangentially migrating cortical interneurons and is likely to act in the cortex to separate the VZ/SVZ and IZ routes.

## Discussion

Although Ascl1 is required for the generation of some cortical interneurons, whether Ascl1 promotes tangential migration has never been revealed. Here, we demonstrate that both Ascl1 and Dlx2 promote tangential migration of neurons derived from either ventral or dorsal telencephalon (Figs 1 and 2). Ascl1 promotes tangential migratory routes through the VZ/SVZ and IZ of the dorsal telencephalon in a *Dlx2*-dependent and a *Dlx2*-independent manner, respectively (Fig. 2). Furthermore, Ascl1 induces the expression of *Ephb2* to confine migratory routes (Figs 4, 5, 6). Ephrin-A5 repels *Ascl1*-expressing neurons *in vitro* and cortical interneurons *in vivo* (Figs 7 and 8), suggesting that the separation of the two migratory routes is maintained by EphB2-Ephrin-A5 signaling. Thus, we propose that Ascl1 induces *Ephb2* in interneurons to avoid *Efna5*-expressing areas and migrate along the VZ/SVZ and IZ routes (Fig. S12).

Several transcription factors regulate cell fate specification and neuronal migration in parallel. However, it is difficult to distinguish whether these transcription factors specify cell fate first and in turn regulate migration versus they play more direct roles in migration. Therefore, it is important to identify target genes of these transcription factors to confirm their roles in migration. Ascl1, Dlx1/2 and Nkx2.1 are important for cell fate specification in the ventral telencephalon. Loss of *Ascl1 , Dlx1*/*2* or *Nkx2 .1* attenuates tangential migration of interneurons^13^,^19^,^23^. Dlx1/2 repress the expression of *Pak3* and *Map2*, two genes encoding regulators of cytoskeletal dynamics. Reduction of *Pax3* and *MAP2* in *Dlx1*/*2* knockout mice rescues the tangential migration defect^15^, suggesting that Pax3 and MAP2 act downstream of Dlx2 in regulating migration. Dlx1/2 also repress the expression of *Neuropilin 2* (*Nrp2*), which encodes a receptor for Semaphorin (Sema)-3A and 3F, to facilitate migration of interneurons toward the dorsal telencephalon^21^. Nkx2.1 induces *ErbB4* in MGE-derived interneurons to enable them to be attracted to the striatum where *Neuregulin 1* is expressed^38^. Nkx2.1 also induces *Ephb1* and *Ephb3* in striatal interneurons to prevent them from entering the dorsal telencephalon^38^. Here, we show that Ascl1 contributes to confine routes of tangential migration in the dorsal telencephalon through inducing *EphB2*. Taken together, these studies all support the model that transcription factors known to specify cell fates may also play a direct role in regulating cell migration.

Previous studies have demonstrated that cortical interneurons generated at different stages select different migratory routes in the dorsal telencephalon^7^. From E12 to E15, cortical interneurons migrate through the MZ and IZ of the dorsal telencephalon^39^,^40^,^41^. After E15, cortical interneurons migrate through the VZ/SVZ, IZ, and MZ. Interestingly, Ascl1 and Dlx1/2 control the generation of interneurons sequentially in the developing telencephalon^13^. In *Ascl1* knockout mice, early-born interneurons at E10.5 and migrating interneurons in the dorsal telencephalon at E12.5 are decreased, suggesting that Ascl1 regulates generation of interneurons at early developmental stage from E10.5 to E12.5^19^,^42^. On the other hand, interneurons born after E15.5 are decreased in *Dlx1*/*2* knockout mice, suggesting that Dlx1/2 regulate interneuron generation at late developmental stages^13^,^43^. These data suggest that Ascl1 and Dlx2 promote different migratory routes in the dorsal telencephalon, which coincides with our findings. Ascl1 promotes tangential migration through the IZ, which is the major migratory route for early-born interneurons (Figs 1F and 2D). Either Ascl1 or Dlx2 promotes tangential migration through the VZ/SVZ route (Figs 1F,G and 2D,E), which is the main migratory route for late-born interneurons. While cortical interneurons are shown to tangentially migrate through the VZ/SVZ, IZ, and MZ of the dorsal telencephalon, the molecular mechanism underlying route selection has never been identified. Thus, our data is the first to provide evidence that Ascl1 and Dlx2 may be key for different migratory behaviors of early- versus late-born cortical interneurons.

Ascl1 has been suggested to act upstream of Dlx1/2 during development of the ventral telencephalon^20^. Interestingly, Ascl1 may regulate the expression of *Dlx1*/*2* negatively or positively^44^,^45^. In the ventral telencephalon, Ascl1 maintains the pool of neural progenitors at least in part by regulating the Notch pathway^42^. Loss of *Ascl1* leads to premature neuronal differentiation and expansion of *Dlx1*/*2* expression domain^19^,^42^. In the developing thalamus, Ascl1 represses *Dlx1*/*2* expression through *Helt*, a bHLH-Orange factor, to specify GABAergic neurons from thalamic progenitors^45^. These findings suggest that Ascl1 acts as a negative regulator for *Dlx1*/*2* expression in some cellular contexts. Here, we found that expression of *Dlx2* was increased by expression of *Ascl1* in P19 cells (Fig. S6A), consistent with previous findings that Ascl1 activates *Dlx1*/*2* in the ventral telencephalon^17^,^20^. Ascl1 and Dlx1/2 have also been demonstrated to act in parallel to regulate neuronal differentiation and migration of LGE-derived olfactory interneurons^46^. Since knockdown of *Dlx2* attenuates the VZ/SVZ migratory route promoted by Ascl1 (Fig. 2F,G), and simultaneous expression of *Ascl1* and *Dlx2* promotes migration through the VZ/SVZ (Fig. S13), our results suggest that Ascl1 induces *Dlx2* to promote tangential migration of interneurons through the VZ/SVZ route of the dorsal telencephalon. In addition, while expression of *Ascl1* induced *Ephb2* in P19 cells, expression of *Dlx2* also increased the level of *Ephb2* mRNA (3.0 ± 0.9 fold of the control, n = 4, Fig. 4). Comparing with expression of *Dlx2* alone, simultaneous expression of *Dlx2* and knockdown of *Ephb2* decreased GFP-positive cells in the VZ/SVZ and increased cells in the IZ and CP (Fig. S14), demonstrating that EphB2 is also critical to confine the VZ/SVZ migratory route promoted by Dlx2. Together, our data suggest that Ascl1 acts upstream or cooperatively with Dlx2 to maintain the tangentially migratory route in the VZ/SVZ, possibly through inducing *Ephb2* cooperatively.

For guiding tangential migration, a chemokine *Cxcl12* is expressed in the dorsal telencephalon to maintain interneurons in a specific migratory route. *Cxcl12* is expressed in the VZ/SVZ to attract cortical interneurons expressing *Cxcr4* and *Cxcr7* receptor genes^47^,^48^,^49^,^50^. Here, we report that migrating interneurons are confined to distinct migratory routes by EphB2/Ephrin-A5 signaling. Thus, chemo-attractive and -repulsive cues may coordinate to guide tangential migration of cortical interneurons.

Here we identify *Ephb2* as a direct target of Ascl1. This is supported by a previous study using ChIP-sequencing in a cellular model of neurogenesis driven by over-expressed *Ascl1*^51^. Previous studies have also reported that Ascl1 regulates radial migration in glutamatergic neurons by inducing a small GTPase gene *Rnd3* and *Cenpj*, a gene encoding a centrosome interacting protein, in the dorsal telencephalon^11^,^16^. Here, we show that Ascl1 is sufficient to promote tangential migration even in the dorsal telencephalon. How does Ascl1 affect different migratory programs in different types of neurons? One possibility is that high level of Ascl1 is capable to activate the tangentially migratory program since *Ascl1* is expressed strongly in the ventral telencephalon but only at a relatively low level in the dorsal telencephalon^52^. Another possibility is that Ascl1 coordinates with other transcription factors or signaling pathways in different cells to determine the migratory program.

In this study, we provide evidence that Ascl1 promotes tangential migration and begin to unravel the mechanisms that underlie this phenomenon. It will be interesting to identify additional Ascl1 target genes that contribute to the regulation of tangential migration. In addition to the links between interneurons and some neurological and psychological diseases^1^,^2^, interneuron transplantation has been demonstrated to relieve seizures, symptoms of Parkinson’s disease and neuropathic pain^53^. A better understanding of the regulation of interneuron migration may contribute to therapeutic approaches for diseases related to interneuron dysfunction.

## Methods

### Animals

Timed-pregnant *Sprague Dawley* (*SD*) rats were obtained from the Laboratory Animal Center in the National Yang-Ming University (NYMU). The *Gad67-GFP*-knock-in (*Gad67-GFP*) mice were provided by Dr. Yanagawa^24^,^54^. All animals were housed in the LAC in NYMU and handled according to the guidelines and animal use protocols approved by the Institutional Animal Care and Use Committee (Approval #: 1031246). *Gad67-GFP* mice were maintained as heterozygous. The sequences of PCR primers used for Genotyping of *Gad67-GFP* mice are: 5′-GGCACAGCTCTCCCTTCTGTTTGC, GCTCTCCTTTCGCGTTCCGACAG, and CTGCTTGTCGGCCATGATATAGACG. The PCR product was a 564-base pair (bp) fragment for the *Gad67-GFP* allele and a 265-bp fragment for the wildtype allele. The embryonic stage was determined as E0.5 for the day when the vaginal plug was observed.

### Plasmids

*sh-LacZ#1 , sh-LacZ#2 , sh-Dlx2#1 , shDlx2#2 , shEphb1#1 , shEphb1#2 , shEphb2#1*, and *shEphb2#2* are in pLKO shRNA expression vector from The RNAi Consortium (TRC) shRNA Library at the Broad Institute. Target sequences of these shRNA constructs are listed in Table S1. Mouse *Neurog2* (NM_009718), *Ascl1* (NM_008553), *Dlx2* (NM_010054), and *Efna5* (NM_207654) were amplified by PCR and inserted into the US2 vector with human Ubiquitin C promoter^55^,^56^.

### In utero electroporation

Surgery for *in utero* electroporation was performed in E15.5 rats and E14.5 mice. The procedure has been described previously^57^. Animals were anesthetized with 2.5% isoflurane (Sigma-Aldrich). An incision was made through the skin and abdominal muscle to expose the viscera. The uterine horns were carefully exposed and placed on wet gauze. DNA solution (2 μg/μL, 0.5 μl total volume) was injected into left lateral ventricle of the telencephalon for each embryo. Forceps electrodes (7 mm in diameter for rats and 5 mm for mice; Harvard Apparatus) were used and five electric pulses separated by 500 ms were transmitted at 50 V for rats and 40 V for mice by an electroporation generator (Harvard Apparatus). The uterine horns were put back into the abdominal cavity after electroporation. Embryos were harvested four days after electroporation for experiments in Figs 1,2,5 and 8, S1, S2, S3, S4, S5, S7, S8, S12 and S13; two days for experiments in Figs 3 and 6, S4 and Supplementary videos.

### Live imaging

Coronal slices of embryonic rat brains were prepared 48 hours after electroporation. Slices were placed on Millicell-CM inserts (Millipore) and incubated at 37 °C with 5% CO_2_ in culture medium that containing 25% Hanks balanced salt solution, 47% basal MEM (Invitrogen), 25% normal horse serum, 100 units/ml penicillin, and 100 μg/ml streptomycin (Invitrogen), and 0.66% glucose. Multiple GFP-positive cells were imaged on an inverted microscope. Time-lapse images were captured by using camera (CoolSNAP HQ; Roper Scientific) at intervals of six minutes for 5 hours and data were analyzed by using MetaMorph software (Molecular Devices).

### Fixation and sectioning

Embryos were perfused with saline and then 4% paraformaldehyde (PFA, Sigma). Brains were post-fixed in 4% PFA overnight, then cryoprotected with 30% sucrose solution and cut frozenly into 60 μm coronal sections with a sliding microtome (Leica). Brain sections were collected and storage in the PBS with 0.02% sodium azide (Sigma) at 4 °C.

### Transfection and differentiation of P19 cells

Mouse P19 cells were maintained in αMEM (Gibco) medium supplement with 100 units/ml penicillin (Invitrogen), 100 μg/ml streptomycin (Invitrogen), 7.5% fetal bovine serum (Hyclone), and 2.5% calf serum (Hyclone). For transfection, cells were plated in 6-well dishes at 80–90% confluence without antibiotics. Lipofectamine 2000 (Invitrogen) was used for transfection according to the manufacturer’s instruction.

### Stripe assay and primary culture of cortical neurons

The procedure of stripe assay has been described previously^58^. A silicon wafer was used to generate a template for poly(dimethylsiloxane) (PDMS) molds with parallel 50 μm stripes separated by 50 μm gaps. PDMS mold was reversibly sealed on 22 × 22 mm^2^ coverslip to form channels for microfluid injection. Microfluid composed of Alexa 549-conjugated donkey anti-guinea pig IgG (1:50, Abcam) and Fc-control (3 μg/ml, Enzo) or Ephrin-A5-Fc (4 μg/ml, R&D system). After injection, microfliud passed through channels by suction. Coverslips were dried for 16 to 18 hours at room temperature and the alternative stripes were formed. Poly-L-lysine (Sigma-Aldrich) and laminin (Sigma-Aldrich) were coated 16 to 18 hours before use.

The protocol for culturing cortical neurons has been described previously^28^. Two days after *in utero* electroporation, the dorsal telencephalon was dissected form E17.5 rat embryos and dissociated by trituration into single cell suspension with fire-polished Pasteur pipettes. 5 × 10^6^ cells were cultured on a coverslip with stripe pattern in L15 medium (Gibco) with N2, B27 (Invitrogen), 30 mM Glucose, 26 mM NaHCO_3_, 100 units/ml penicillin, and 100  μg/ml streptomycin (Invitrogen). Cells were cultured for 16 to 18 hours and fixed in 4% PFA. At least 150 GFP-positive neurons were counted for each group.

### Immunofluorescence

Sections or coverslips with cells were washed with Tris buffered saline (TBS) and incubated in blocking buffer (1% glycine, 0.4% Triton X-100, 3% BSA, 0.1% sodium azide and 10% normal goat serum in TBS) for one hour at room temperature. Sections or coverslips were incubated in the primary antibodies in species-appropriate combinations for 24 hours at 4 °C. Primary antibodies and dilution used in our experiments included rabbit anti-GFP (Catalog: A-11122, 1:1500, Invitrogen), rat anti-GFP (Catalog: GF090R, 1:2000, Nacalai), mouse anti-MAP2 (Catalog: ab11267, 1:200, Abcam), Goat anti-EphB2 (Catalog: AF467, 1:250, R&D systems), rabbit anti-Ephrin-A5 (Catalog: ab70114, 1:250, abcam) and rabbit anti-RFP (Catalog: ab62341, 1:250, Abcam). After incubation with primary antibodies, sections or coverslips were washed with TBS and incubated with secondary antibodies in blocking buffer at room temperature for two hours. Secondary antibodies included Alexa 488, 546, 633 conjugated goat anti-mouse, anti-rat, anti-rabbit IgG (1:500; Abcam). Sections or coverslips were then washed with TBS and mounted with ProLong Gold anti-fade (Invitrogen). All images were taken by Zeiss LSM700 confocal microscopy. The volume, migratory distance, and the average fluorescent intensity were estimated by using ImageJ (U. S. National Institutes of Health).

### RNA extraction and Quantitative RT-PCR

P19 cells in 6-well plates were transfected with 1 μg of US2-puro (puromycin-resistant gene) and 3 μg of various expression constructs. Transfected cells were selected in puromycin (15 μg/ml) for eight hours. Total RNA was extracted one day after transfection by using the RNeasy Mini Kit (QIAGEN). cDNA was prepared from 3  μg of total RNA by using SuperScript III Reverse Transcriptase (Invitrogen). 10 μl of PCR mix contains 1× FastStart Universal SYBR Green Master (Roche), 20 μM forward primer, 20 μM reverse primer (primer sequences are listed in Table S2), and 2.5 ng/μl cDNA template. Quantitative PCR was performed with StepOnePlus™ Real-Time PCR System (Applied Biosystems) and analyzed with the StepOnePlus V2.3 software. *TATA-box binding protein* (*Tbp*) was used as a control. MIQE is in Table S3 and raw data of qPCR are available in Supplementary spreadsheet I.

### Chromatin Immunoprecipitation (ChIP)

The procedure of ChIP has been described previously^59^. P19 cells were transfected with *Ascl1* expression vectors and cultured for one day. Genomic DNA from 1 × 10^7^ cells was used for precipitation with each antibody. Cellular contents were cross-linked with 1% formaldehyde for five minutes at room temperature and stopped by incubation in 0.125 M of glycine for 10 minutes at room temperature. Fixed cells were rinsed twice with PBS and re-suspended by using Trypsin-EDTA (Invitrogen). Cell lysate was then sonicated for 5 min and centrifuged at 14000 rpm for 10 min. The cleared supernatant was used immediately for IP. Sonicated DNA was blocking for one hour at room temperature and incubated with mouse anti-Ascl1 (BD Biosciences) or rabbit non-immune serum IgG (Alpha Diagnostic International) for overnight. Precipitated materials were eluted by elution buffer (SDS 1%, 0.1 M NaHCO3). DNA was reverse-cross-linked in 5M NaCl and treated with RNase and proteinase K. DNA was purified by using phenol-chloroform. PCR primer sequences for amplifying putative Ascl1 binding fragments were listed in the Table S3. Raw data of qPCR are available as Supplementary spreadsheet II.

### Statistical analysis

Statistical analysis was performed by using SPSS (IBM). Two-tailed Student’s t test was used for comparison between two groups and ANOVA with Tukey’s-HSD post hoc test was used for comparison among three or more groups. Chi-square analysis was used for the analysis in Fig. 6.

## Additional Information

**How to cite this article**: Liu, Y.-H. *et al*. Ascl1 promotes tangential migration and confines migratory routes by induction of *Ephb2* in the telencephalon. *Sci. Rep.* **7**, 42895; doi: 10.1038/srep42895 (2017).

**Publisher's note:** Springer Nature remains neutral with regard to jurisdictional claims in published maps and institutional affiliations.

## Supplementary Material

Supplementary Tables and Figures

Supplementary Video I

Supplementary Video II

Supplementary Spreadsheet II

Supplementary Spreadsheet I
