# Supplementary material for: Ascl1 promotes tangential migration and confines migratory routes by induction of Ephb2 in the telencephalon
Source: Sci Rep. 2017 Mar 9;7:42895. doi: 10.1038/srep42895 (PMC5343589; doi:10.1038/srep42895)
Supplement: Supplementary Tables and Figures [file srep42895-s1.pdf]

**Ascl1 promotes tangential migration and confines migratory routes by induction of Ephb2 in the telencephalon**

Yuan-Hsuan Liu, Jin-Wu Tsai, Jia-Long Chen, Wan-Shan Yang, Pei-Ching Chang, Pei-Lin Cheng, David L. Turner, Yuchio Yanagawa, Tsu-Wei Wang, Jenn-Yah Yu

# Supplementary information

**Table S1. The primer sequences used for quantitative RT-PCR**

| **Gene** | **Forward primer (5'->3')** | **Reverse primer (5'->3')** |
|---|---|---|
| *Epha3* | TGGCTCCTTGGACAGTTTCT | TTCCCACAAGCTGGATGACT |
| *Epha4* | AGGTGTCTGACTTTGGCATGT | CAGTCCACCGGATAGGAATC |
| *Epha5* | TTGCGAAGGAGATTGAAGC | GCAAACTTCACCAAATTCACC |
| *Ephb1* | CAAGACTGTGGCAACCATCA | AGTCTGGGATAGAGCGGTCA |
| *Ephb2* | TAGACATGCCTTGCACAACC | GTCTCGTTGACGCTGGAGAT |
| *Efna5* | GCCAGGCCGAGAGTATTTC | CAGGACCTTCTTCCGTTGTC |
| *Efnb1* | AGTGGCTTGTGGCTATGGTC | GGGTTAAGAGAGCTCCAGGAC |
| *Efnb2* | GGTCAAGGCTGCTTGCTG | GCATTATCTATTTCCTTCCTGCAT |
| *Efnb3* | TGGAACTCGGCGAATAAGAG | CCCCGATCTGAGGATAAAGC |
| *Dlx2* | GCCTCACCCAAACTCAGGT | AGGCACAAGGAGGAGAAGC |
| *Tbp* | CGGTCGCGTCATTTTCTC | GGGTTATCTTCACACACCATGA |

**Table S2**. **Target sequences for *Dlx2*, *Ephb1*, and *Ephb2* of shRNA expression constructs**

| **shRNA expression construct** | **Target sequence (5'->3')** |
|---|---|
| shDlx2#1 | CTTGAGCCTGAAATCCGAATA |
| shDlx2#2 | CCGATCCAAGTTCAAGAAGAT |
| shEphB1#1 | CCATCGCCTACCGCAAGTTTA |
| shEphB1#2 | CCGGCCTGTCATGATCATTAC |
| shEphB2#1 | CCTTAGACATGCCTTGCACAA |
| shEphB2#2 | CAAGACGTAATCAACGCCATT |

**Table S3**. **The primer sequences used for PCR after ChIP**

| Promotor/Enhancer | Forward primer (5’->3’) | Reverse primer (5’->3’) |
|---|---|---|
| *Dll1* | GCGTGGCTGTCATTAAGG | GGTGCTGTCTGCATTACC |
| | | |
| *Ephb1* (B1R1) | AGCAGGGTTCTTTTTGGATG | TTTTGCTGTCCCTCTGATCC |
| *Ephb1* (B1R2) | CGGGGTTACAGAGGACAGAA | CAAGCACACCCAGCAAAAC |
| *Ephb1* (B1R3) | CAGGGTGGGGAATCAATGTA | GTCAGAAGCTGTGTGGCAGA |
| *Ephb1* (B1R4) | GCCCTACAGTGGATGCTCAG | GCATGCATCTGTGGAGTGAG |
| *Ephb1* (B1NE) | CCATCCATCCATCCATCAGT | TTCATCCCTCCAGCCTCTAA |
| | | |
| *Ephb2* (B2R1) | GGTCTGGATCCAAAGCTCAC | GAAAGATGATCACGGGTTCC |
| *Ephb2* (B2R2) | AAGGGATGAGTGTGGGTGTC | GGCTGTTCACGTTTCCAGAT |
| *Ephb2* (B2R3) | CCCCAGGGGATAGAAAGAAA | CAGACAAACCAAGGGGCTTA |
| *Ephb2* (B2R4) | TTCTGTTCTGCCTCCTTTGC | CAAGGCCCATCAGACAGACT |
| *Ephb2* (B2R5) | CATCCGAAGGGACTCAAAAG | CAGCAGAATGAGCCATCCA |
| *Ephb2* (B2NE) | AAATGGAACAAGGGGTTGTG | CCGCACATTGTATTTCTCCTC |

**Table S4**. **Minimum Information for Publication of qPCR Experiments**

| Item to Check | Importance | Checklist | Description |
|---|---|---|---|
| **Experimental design** | | | |
| Definition of experimental and control groups | E | Yes | Control:US2<br>Experimental group: Neurog2, Ascl1, and Dlx2 |
| Number within each group | E | Yes | N=4 |
| Assay carried out by core lab or investigator's lab? | D | Yes | investigator's lab |
| **Sample** | | | |
| Description | | | P19 cell line |
| Volume/mass of sample processed | D | Yes | $5X10^6$ cells/well |
| Microdissection or macrodissection | E | Yes | Cells were lysed by lysis buffer with 1% 2-*mercaptoethanol* |
| Processing procedure | E | Yes | |
| If frozen - how and how quickly? | E | N/A | |
| If fixed - with what, how quickly? | E | N/A | |
| Sample storage conditions and duration | E | Yes | Samples stored at -80°C and proceed to analyzed within one week. |
| **Nucleic Acid Extraction** | | | |
| Procedure and/or instrumentation | E | Yes | All procedures were followed the guidance of QIAGEN's RNeasy Mini Kit (Catalog; 74014). |
| Name of kit and details of any modifications | E | Yes | |
| Source of additional reagents used | D | Yes | Add 4μL RQ1 Dnase (10U/μL, Promega) into reaction solution at 37°C for 30 mins. |
| Details of DNase or RNAse treatment | E | Yes | |
| Nucleic acid quantification | E | Yes | We used Nanodrop ND-1000 to quantify our extracted RNA. All quantification methods were followed the user's manual. The extracted products with the ratio of A260/A80 around 2.0 were preceding the following experiments. |
| Instrument and method | E | Yes | |
| Purity (A260/A280) | D | Yes | |
| Yield | D | Yes | |
| RNA integrity method/instrument | E | Yes | We used electrophoresis to check the integrity of extracted RNA. |
| RIN/RQI or Cq of 3' and 5' transcripts | E | No | |
| Electrophoresis traces | D | Yes | |
| Inhibition testing (Cq dilutions, spike or other) | E | No | |
| **Reverse Transcription** | | | |
| Complete reaction conditions | E | Yes | |

| Component | |
|---|---|
| Total RNA<br>Oligo$(dT)_{12-18}$ primer, 0.5μg/μL<br>25mM dNTP<br>100mM DTT<br>5X First-strand buffer | 2 μg<br>1 μL<br>0.4 μL<br>1 μL<br>4 μL |

| | | | |
|---|---|---|---|
| (250 mM Tris-HCl (pH 8.3), 375 mM KCl, 15 mM MgCl2)<br>RNasin® Ribonuclease Inhibitor, 40U/μL<br>Nuclease-free water | | | <br>1 μL<br>Up to 20 μL |
| Amount of RNA and reaction volume | E | Yes | 2 μg |
| Priming oligonucleotide and concentration | E | Yes | Oligo(dT)$_{12-18}$ (Invitrogen), 0.5 μg/μL |
| Reverse transcriptase and concentration | E | Yes | Superscript® III Reverse transcriptase (Invitrogen), 200U/μL |
| Temperature and time | E | Yes | cDNA was synthesized at 50°C for 120 minutes. We Inactivate the reaction by heating at 70°C for 15 minutes. |
| Manufacturer of reagents and catalogue numbers | D | Yes | RNasin® (Promega): N2111 |
| Cqs with and without RT | D | No | |
| Storage conditions of cDNA | D | Yes | Storage the cDNA at -20°C |
| **qPCR Target Information** | | | |
| If multiplex, efficiency and LOD of each assay | E | N/A | |
| Sequence accession number | E | Yes | EphA3: NM_010140<br>EphA4: NM_007936<br>EphA5: NM_007937<br>EphB1: NM_001168296<br>EphB2: NM_001290753<br>Ephrin-B1: NM_010110<br>Ephrin-B2: NM_010111<br>Ephrin-B3: NM_007911<br>Dlx2: NM_010054 |
| Location of amplicon | D | N/A | |
| Amplicon length | E | Yes | 130-150 b.p. |
| In silico specificity screen (BLAST, etc) | E | Yes | BLAST |
| Pseudogenes, retropseudogenes or other homologs? | D | Yes | Non-detected in the BLAST |
| Sequence alignment | D | Yes | Alignment has been checked during the specificity screen in the BLAST. |
| Secondary structure analysis of amplicon | D | No | |
| Location of each primer by exon or intron (if applicable) | E | Yes | |
| What splice variants are targeted? | E | N/A | |
| **qPCR Oligonucleotides** | | | |
| Primer sequences | E | Yes | See sup. Table S1 and S2 |
| RTPrimerDB Identification Number | D | N/A | |
| Probe sequences | D | N/A | |
| | | | |
| Location and identity of any modifications | E | N/A | |
| Manufacturer of oligonucleotides | D | Yes | Mission Biotech |

| | | | |
|---|---|---|---|
| Purification method | D | Yes | HPLC |
| qPCR PROTOCOL | | | |
| Complete reaction conditions | E | Yes | |
| Component | | | Amount |
| 2X SYBR Green Master mix (Roche)<br>20 µM Forward primer<br>20 µM Reverse primer<br>cDNA (1:10 diluted from stock cDNA)<br>Nuclease-free water<br>total | | | 6 µL<br>0.6 µL<br>0.6 µL<br>2 µL<br>2.8 µL<br>12 µL |
| Reaction volume and amount of cDNA/DNA | E | Yes | 1:10 diluted from stock cDNA, used 2 µL/tube |
| Primer, (probe), Mg++ and dNTP concentrations | E | Yes | |
| Polymerase identity and concentration | E | Yes | 2X SYBR Green Master mix (04913850001, Roche) |
| Buffer/kit identity and manufacturer | E | Yes | 2X SYBR Green Master mix (Roche) |
| Exact chemical constitution of the buffer | D | NO | Manufactures proprietary |
| Additives (SYBR Green I, DMSO, etc.) | E | N/A | |
| Manufacturer of plates/tubes and catalog number | D | Yes | Gunster, catalog: MB-q100 |
| Complete thermocycling parameters | E | Yes | 95°C 15 for 15 sec, then 60°C for 1 min. Total 40 cycles. |
| | | | |
| Reaction setup (manual/robotic) | D | Yes | Manual setup |
| Manufacturer of qPCR instrument | E | Yes | StepOnePlus™ Real-Time PCR System (Applied Biosystems) |
| **qPCR Validation** | | | |
| Evidence of optimization (from gradients) | D | No | |
| Specificity (gel, sequence, melt, or digest) | E | Yes | Melt curve.<br>See Supplementary spreadsheet I |
| For SYBR Green I, Cq of the NTC | E | Yes | NTC was undetected in each experiment. |
| Standard curves with slope and y-intercept | E | Yes | See Supplementary spreadsheet I |
| PCR efficiency calculated from slope | E | Yes | See Supplementary spreadsheet I |
| Confidence interval for PCR efficiency or standard error | D | N/A | |
| r2 of standard curve | E | Yes | See Supplementary spreadsheet I |
| Linear dynamic range | E | N/A | |
| Cq variation at lower limit | E | N/A | |
| Confidence intervals throughout range | D | N/A | |
| Evidence for limit of detection | E | N/A | |
| If multiplex, efficiency | E | N/A | |

| | | | |
|---|---|---|---|
| and LOD of each assay | | | |
| **Data Analysis** | | | |
| qPCR analysis program (source, version) | E | Yes | **All data were processing by StepOnePlus$^{TM}$ V2.3** software (Applied Biosystems). |
| Cq method determination | E | Yes | |
| Outlier identification and disposition | E | Yes | |
| Results of NTCs | E | Yes | NTC was undetected in each experiment. |
| Justification of number and choice of reference genes | E | Yes | TATA biding protein (TBP) was selected as the reference gene. We normalized target's Cq to reference's Cq to get delt-Cq. |
| Description of normalization method | E | Yes | |
| Number and concordance of biological replicates | E | Yes | N=4 |
| Number and stage (RT or qPCR) of technical replicates | E | Yes | N=3 |
| Repeatability (intra-assay variation) | E | Yes | We use SPSS V.16 (IBM) to analyze our results. The Statistical significance was checked by ANOVA with Tukey's-HSD post hoc test. |
| Reproducibility (inter-assay variation, %CV) | D | No | |
| Power analysis | D | No | |
| Statistical methods for result significance | E | Yes | |
| Software (source, version) | E | Yes | |
| Cq or raw data submission using RDML | D | N/A | The raw Cq was listed in the Supplementary spreadsheet I. |

E: Essential information, D: Desirable information, N/A: Not applicable

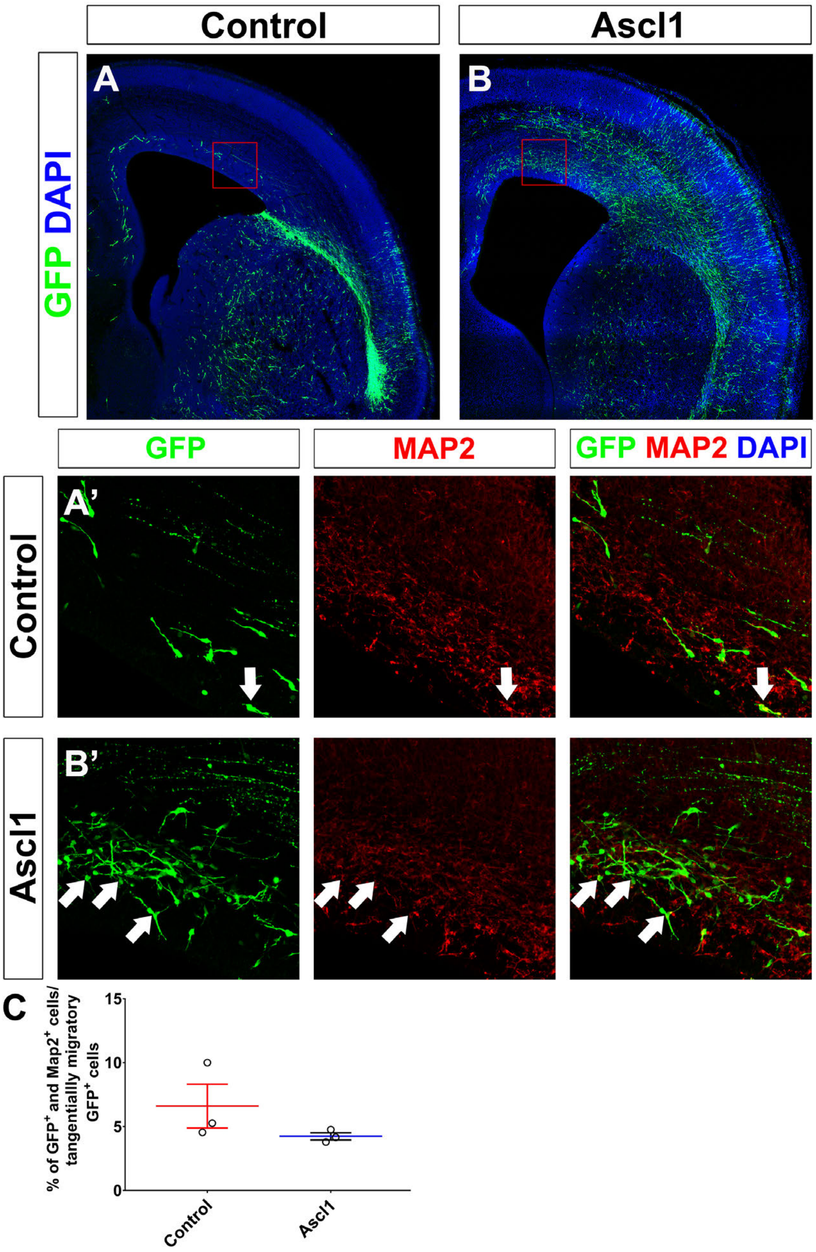


**Figure S1. Overexpression of *Ascl1* in the ventral telencephalon does not promote premature neuronal differentiation.** Four days after electroporation, brains of E19.5 rats were dissected and sectioned in the coronal plane. Electroporated cells were labeled with anti-GFP in green. Differentiating and differentiated neurons were labeled with anti-MAP2 in red. Nuclear DNA was stained with DAPI in blue. (A-B) Confocal images; a red square indicates the zoomed area for (A', B'). (A', B') In both the control and Ascl1 groups, some GFP-positive cells were distributed in the VZ/SVZ of the dorsal telencephalon. A few GFP-positive cells were positive for MAP2 (indicated by white arrows). Length of the scale bar is 120 μm in (A-B), and 50 μm in (A'-B'). (C) Quantification of GFP and MAP2 double-positive cells over total GFP-positive cells in the dorsal telencephalon. Data were presented as mean±SEM with all data points and analyzed by Student's t-test, n=3.

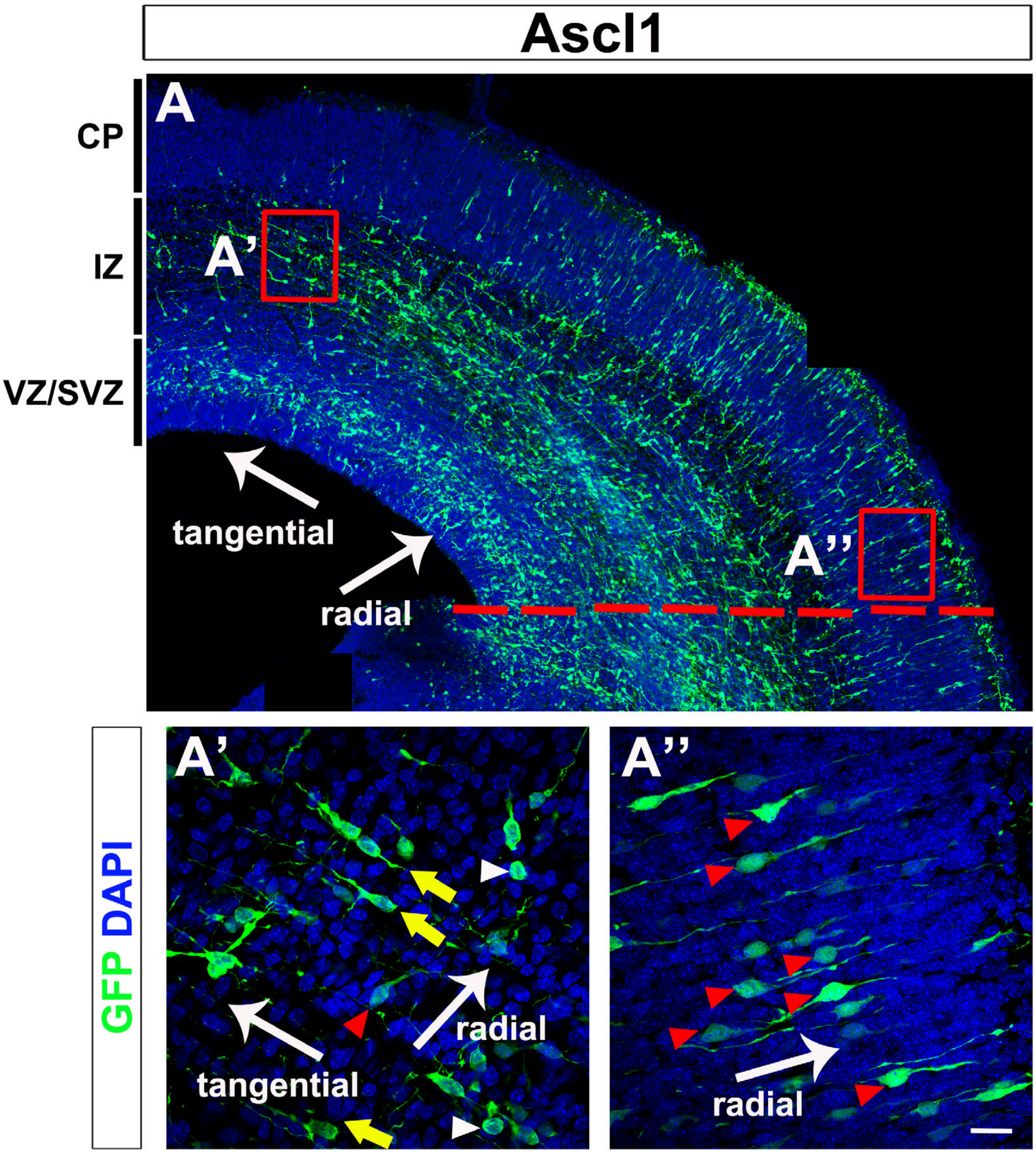


**Figure S2. Categorization of GFP-positive cells based on the orientation of their leading processes.** Four days after electroporation of *Ascl1* and *GFP* expression constructs, brains of E19.5 rats were dissected and sectioned in the coronal plane. Electroporated cells were labeled with anti-GFP in green; nuclear DNA was stained with DAPI in blue. (A) Zoomed areas for the IZ (A') and the CP (A'') were indicated by red squares. GFP-positive cells with leading processes horizontal to the boundary of the lateral ventricle were categorized as tangentially migrating cells (yellow arrows). GFP-positive cells with leading processes vertical to the boundary of the lateral ventricle were categorized as radially migrating cells (red arrowheads). GFP-positive cells without leading processes or the orientation of their leading processes were not clear were categorized as other cells (white arrowheads). (A') GFP-positive cells in the IZ were tangentially migrating cells, radially migrating cells, and other cells. (A'') Most GFP-positive cells in the CP were radially migrating cells. The length of scale bar is 20 μm in (A') and (A'').

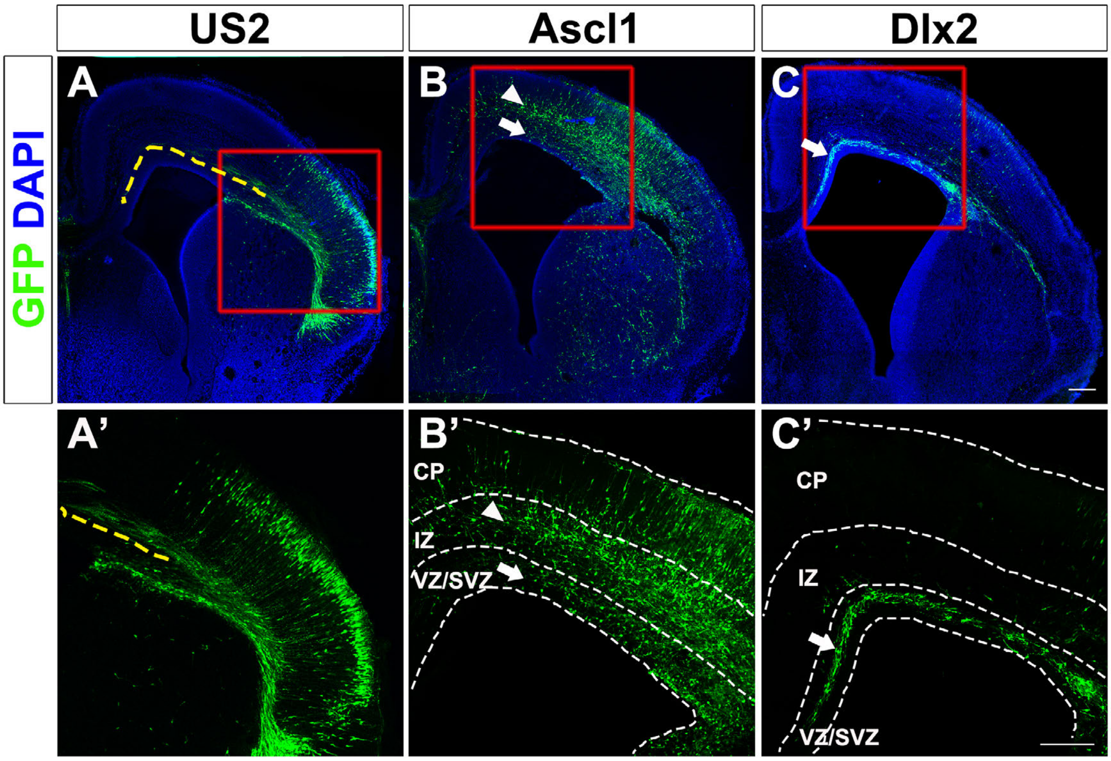


**Figure S3. Overexpression of *Ascl1* and *Dlx2* in the dorsal telencephalon induces ectopically tangential migration in mice.** Four days after electroporation, brains of E18.5 mice were dissected and sectioned in the coronal plane. Electroporated cells were labeled with anti-GFP in green; nuclear DNA was stained with DAPI in blue. (A'-C') are zoomed regions of (A-C) indicated by red squares. (A) In the control group. GFP-positive cells were distributed near or above the electroporated area and many of them extended processes radially. GFP-positive axons extending to the contralateral side were labeled by yellow dashed line. (B) In Ascl1 group, many GFP-positive cells were distributed in the VZ/SVZ (white arrows) and IZ (white arrowheads) dorsomedial to the electroporated site. (C) In Dlx2 group, many GFP-positive cells were distributed in the VZ/SVZ (white arrows) dorsomedial to the electroporated site. The length of scale bar is 100 μm.

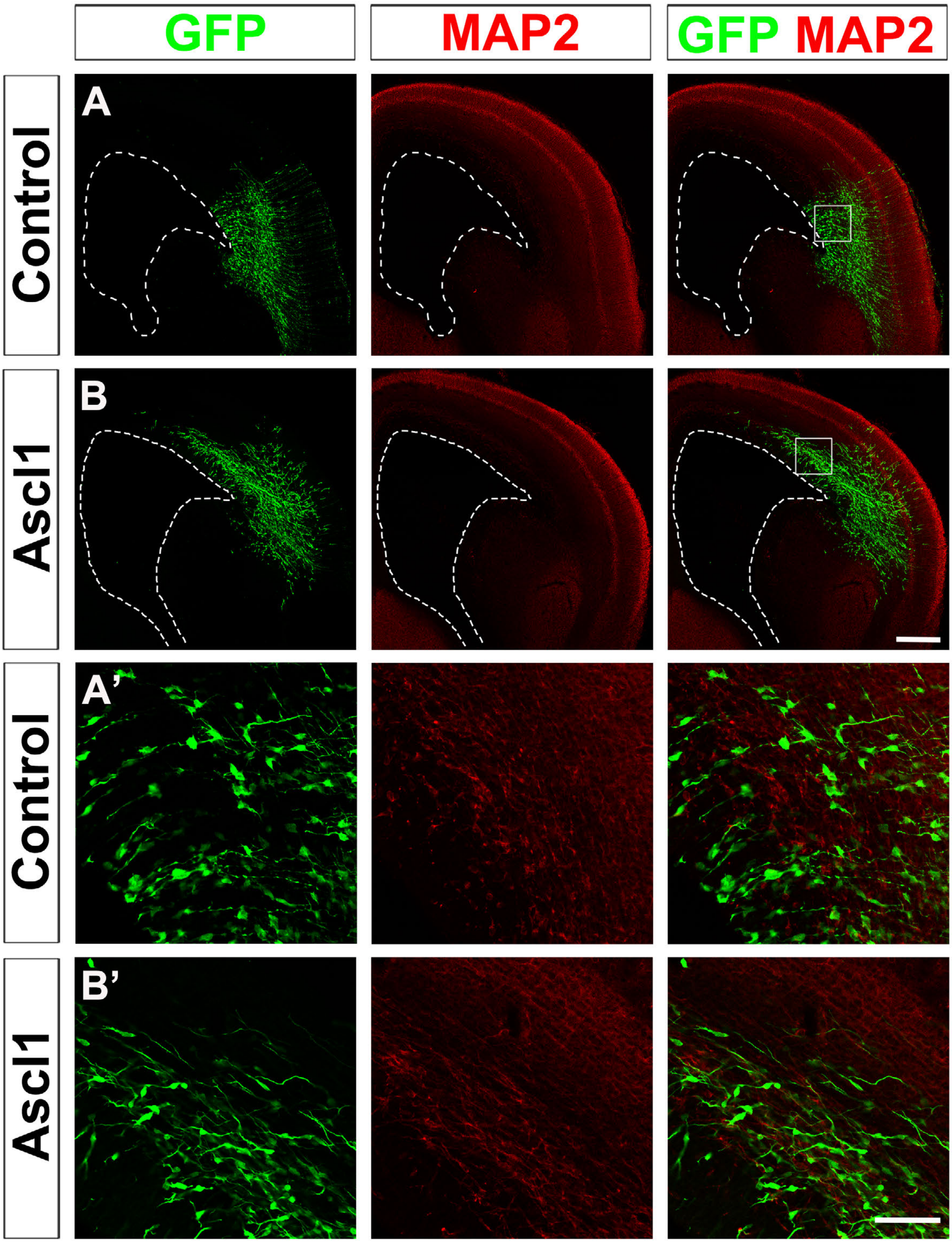


**Figure S4. Overexpression of *Ascl1* in the dorsal telencephalon does not promote premature neuronal differentiation.** Two days after electroporation, brains of E17.5 rats were dissected and sectioned in the coronal plane. Electroporated cells were labeled with anti-GFP in green. Differentiated neurons in the CP were labeled with anti-MAP2 in red. (A, B) Confocal images of tissue sections; a white square indicates the area for (A', B'). (A', B') In both the control and Ascl1 groups, no MAP2 and GFP-double positive cells were detected in the IZ and VZ/SVZ of the dorsal telencephalon. Length of the scale bar is 120 μm in (A-B), and 50 μm in (A'-B').

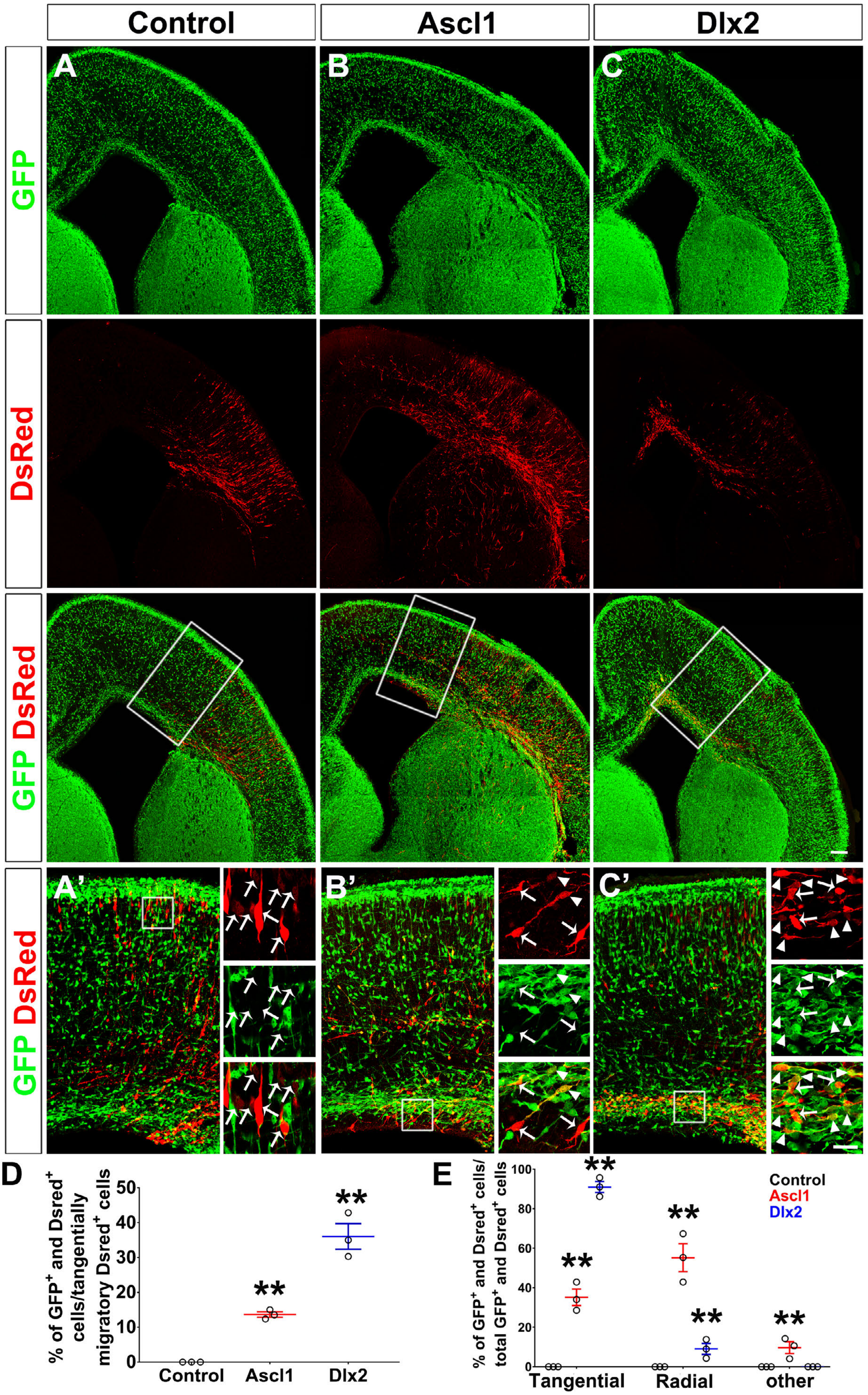
Control
Ascl1
Dlx2
A
B
C
GFP
DsRed
GFP DsRed
A'
B'
C'
D
% of GFP+ and Dsred+ cells/tangentially migratory Dsred+ cells
50
40
30
20
10
0
**
**
Control
Ascl1
Dlx2
E
% of GFP+ and Dsred+ cells/ total GFP+ and Dsred+ cells
100
80
60
40
20
0
Tangential
Radial
other
Control
Ascl1
Dlx2

**Figure S5. Overexpression of *Ascl1* and *Dlx2* in the dorsal telencephalon induces ectopic GABAergic interneurons in *Gad67-GFP* mice.** Four days after electroporation, brains of E18.5 *Gad67-GFP* mice were dissected and sectioned in the coronal plane. Electroporated cells were labeled with anti-DsRed; nuclear DNA was stained with DAPI in blue. (A'-C') were zoomed regions of (A-C) indicated by white squares. (A-A') In the control group, most DsRed-positive cells extend radial processes. None of the DsRed-positive cells were *Gad67*-GFP-positive (white arrows). (B) In Ascl1 group, many DsRed-positive cells were distributed in the VZ/SVZ and IZ dorsomedial to the electroporated site. (C) In Dlx2 group, many DsRed-positive cells were distributed in the VZ/SVZ dorsomedial to the electroporated site. (B', C') Some DsRed-positive cells were *Gad67*-GFP-positive (white arrowheads); some DsRed-positive cells were *Gad67*-GFP-negative (white arrows). The length of scale bar is 100 μm. (D) Quantification of *Gad67*-GFP and Dsred double-positive cells over total DsRed-positive cells in the dorsal telencephalon. Data are presented as mean±SEM with all data points. Student's t-test, n=3. *: $p<0.05$; **: $p<0.01$. (E) *Gad67*-GFP and Dsred double-positive cells were categorized into radial, tangential, or other types according to the orientation of their leading processes. Data are presented as mean±SEM with all data points. Student's t-test, n=3. *: $p<0.05$; **: $p<0.01$.

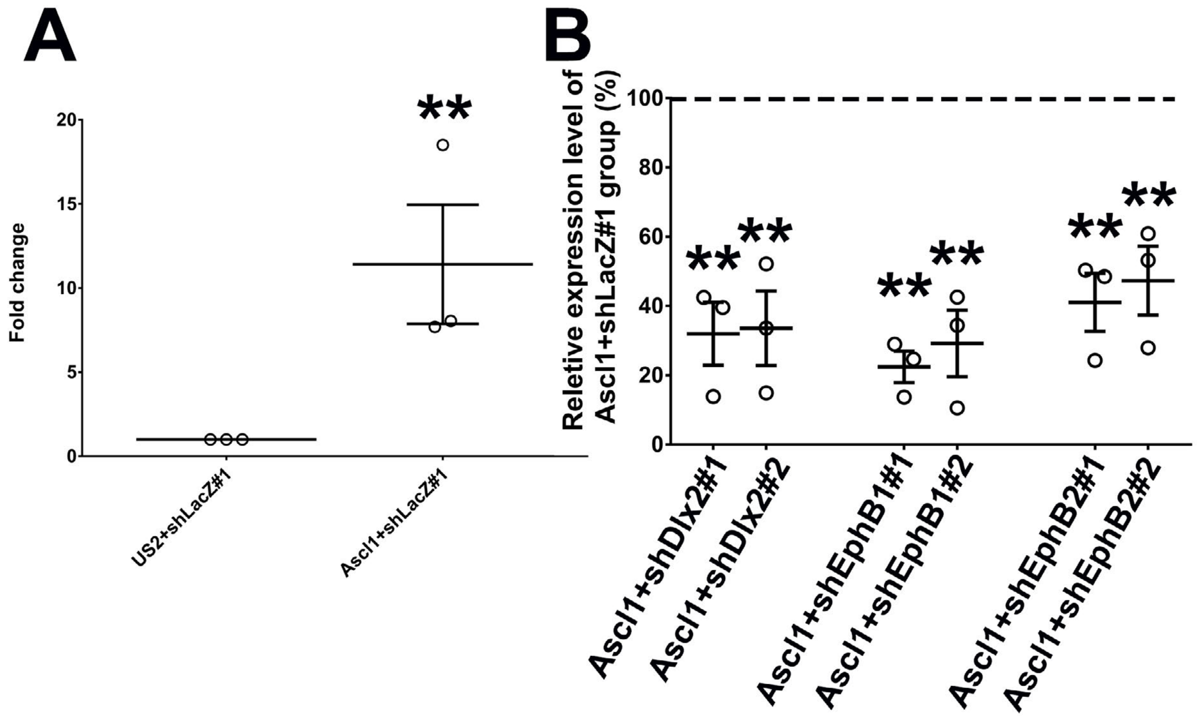


**Figure S6. Efficient knockdown of *Dlx2*, *Ephb1*, and *Ephb2* in *Ascl1*-expressing P19 cells.** Total RNA of P19 cells was extracted two days after transfection for quantitative RT-PCR. (A) P19 cells were transfected with control (*US2*) or *Ascl1* expression construct. shLacZ#1 was used as a control shRNA expression construct. The level of *Dlx2* mRNA was increased in the *Ascl1*-expressing P19 cells comparing with that in the control group. (B) P19 cells were transfected with *Ascl1* and shLacZ, shDlx2, shEphB1 or shEphB2. We used two shRNA expression constructs to target each gene (#1 and #2). The mRNA level of *Dlx2*, *Ephb1*, or *Ephb2* in Ascl1+shLacZ#1 group was used for normalization (100%, indicated by black dash line). Expression of *Dlx2*, *Ephb1*, or *Ephb2* was decreased upon transfection of shDlx2, shEphB1 or shEphB2 vectors, respectively. Data are presented as mean±SEM with all data points and analyzed by using Student's test, n=3; **: $p<0.01$.

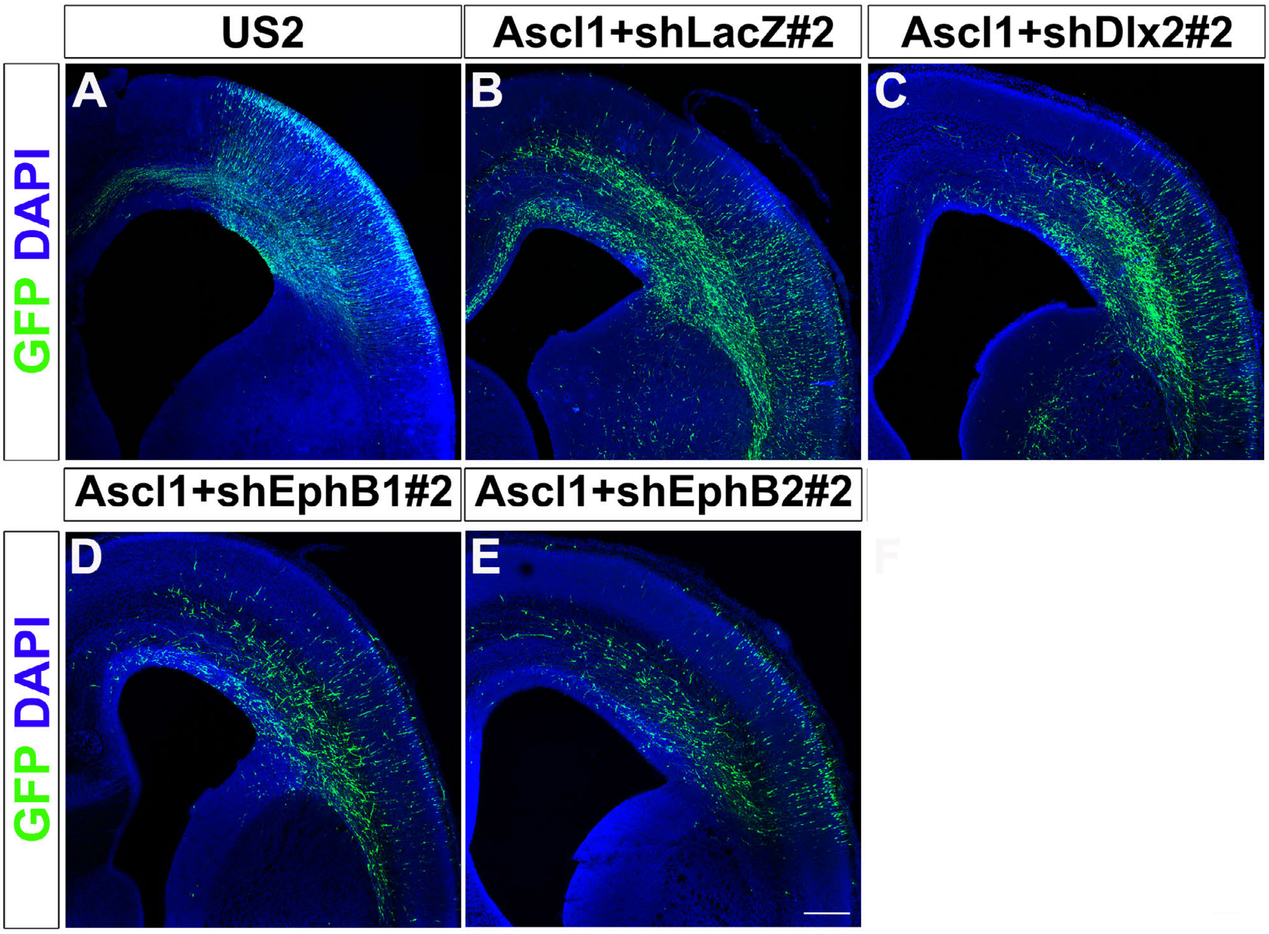


**Figure S7. Knockdown of *Dlx2*, *Ephb1*, or *Ephb2* disrupts tangential migration promoted by Ascl1.** Four days after electroporation to the dorsal telencephalon, brains of E19.5 rats were dissected and sectioned in the coronal plane. Electroporated cells were labeled with anti-GFP in green; nuclear DNA was stained with DAPI in blue. Additional shRNA expression constructs were used to knock down *LacZ*, *Dlx2*, *Ephb1*, and *Ephb2* in the dorsal telencephalon. Similar to shDlx2#1, EphB1#1, or EphB2#1 in Fig. 2 and 4, electroporation of shDlx2#2, EphB1#2, or EphB2#2 affected distribution of GFP-positive cells. Length of the scale bar is 120 μm. US2 control group: n=6. All other groups: n=3.

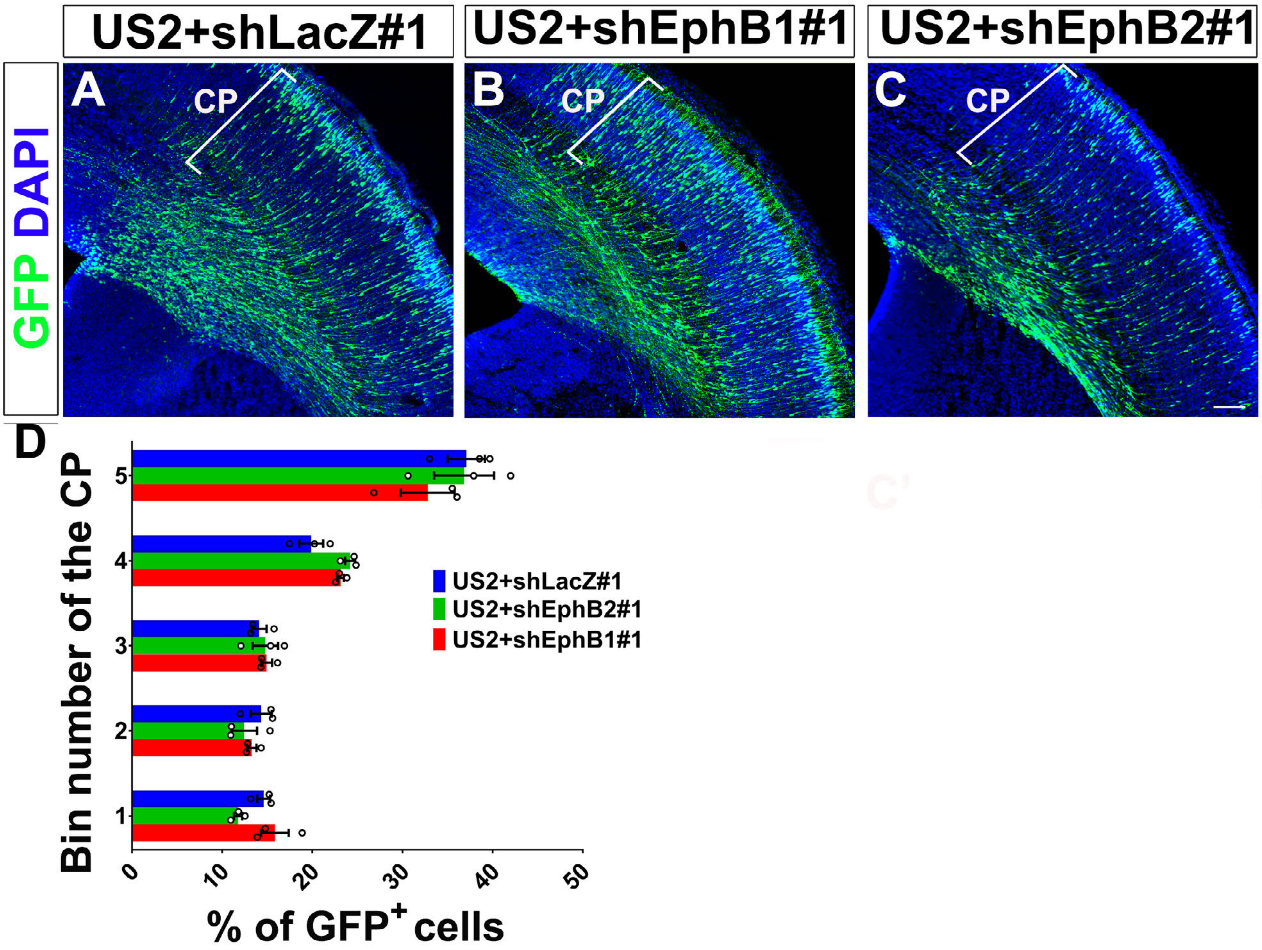


**Figure S8. Knockdown of *Ephb1* or *Ephb2* does not attenuate radial migration.** Four days after electroporation to the dorsal telencephalon, brains of E19.5 rats were dissected and sectioned in the coronal plane. Electroporated cells were labeled with anti-GFP in green; nuclear DNA was stained with DAPI in blue. No *Ascl1* expression vector was transfected. US2 and shLacZ#1 vectors were used as controls. (A-C) The distributions of GFP-positive cells in the CP were similar among US2+shLacZ#1, US2+shEphB1#1, and US2+shEphB2#1. Length of the scale bar is 100 μm. (D) The CP was divided equally into 5 bins and GFP-positive cells in each bin were counted. The distributions of GFP-positive cells in the CP were similar among three groups. Data are presented as mean±SEM with all data points and analyzed by using Student's test, n=3.

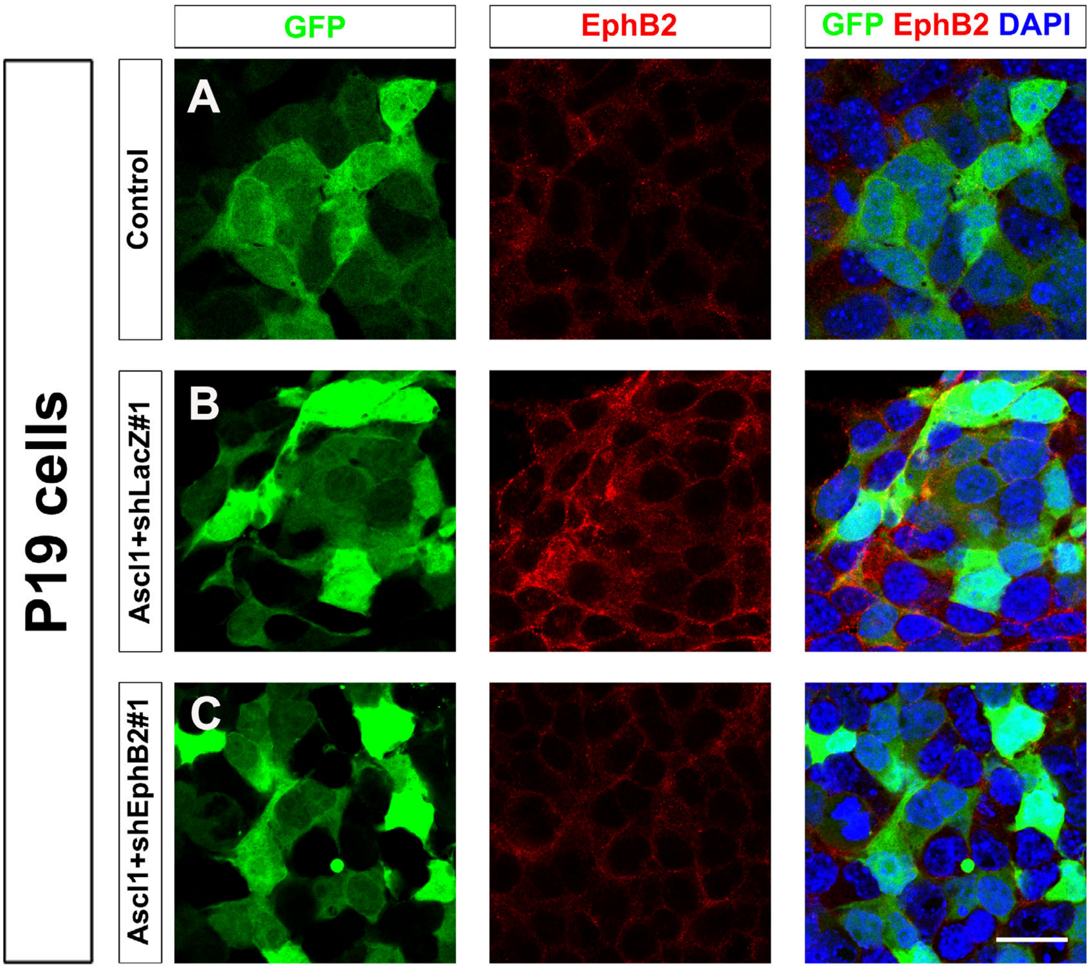


**Figure S9. Overexpression of *Ascl1* increases the level of EphB2 in P19 cells.** P19 cells were transfected with expression constructs and fixed for immunofluorescence two days after transfection. A GFP expression construct was co-transfected to indicate transfected cells. Cells were labeled with anti-EphB2 in red. Nuclear DNA was stained with DAPI in blue. (A) A low level of EphB2 was detected in P19 cells. (B) Expression of *Ascl1* increased the immunofluorescent staining signal of EphB2. (C) Knockdown of *Ephb2* substantially decreased the immunofluorescent staining signal of EphB2 in *Ascl1*-expressing cells. The length of scale bar is 20 μm.

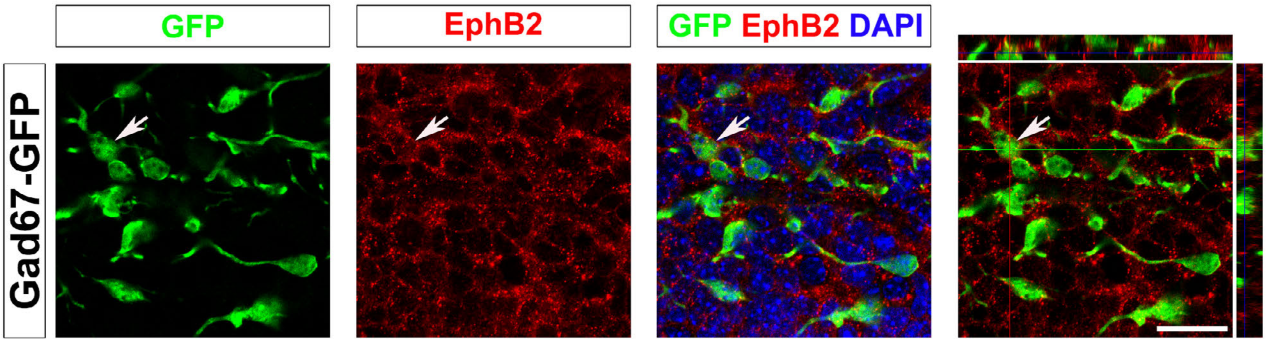


**Figure S10. EphB2 is detected in cortical interneurons.** Brains of E18.5 *Gad67-GFP* mice were dissected and sectioned in the coronal plane. The brain sections were labeled with anti-GFP in green and anti-EphB2 in red. Nuclear DNA was stained with DAPI in blue. The confocal image was taken from the VZ/SVZ region of dorsal telencephalon. A stacked image shows that a GFP-positive cell is also EphB2-positive (indicated by arrows). Length of the scale bar is 20 μm.

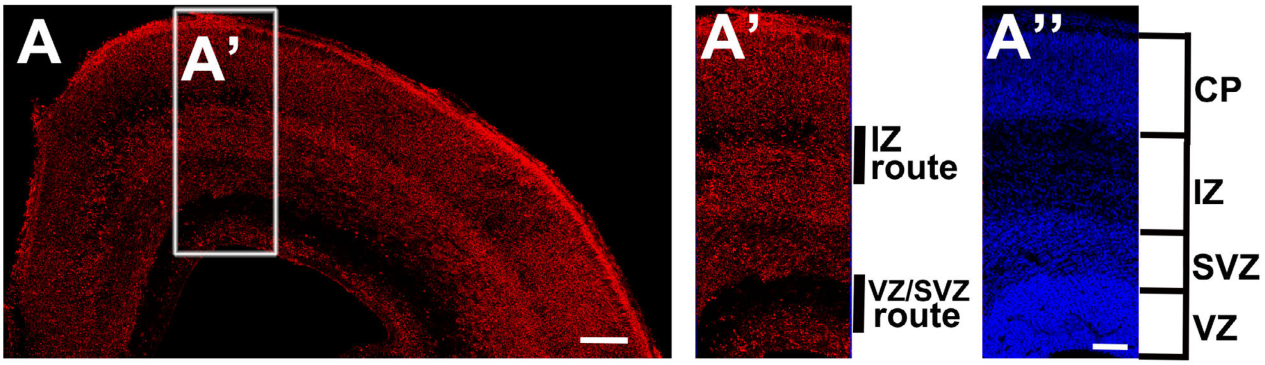


**Figure S11. Ephrin-A5 is detected in the dorsal telencephalon.** E19.5 rat brains were dissected and sectioned in the coronal plane. Anti-Ephrin-A5 signal was labeled in red; nuclear DNA was stained with DAPI in blue. A white square indicates the zoom-in areas for A' and A". The IZ and VZ/SVZ migratory routes are indicated in A'; the CP, IZ, SVZ and VZ are indicated in A". Four stripes are positive for Ephrin-A5: deep VZ, upper SVZ, deep IZ and CP. Length of the scale bar is 100 μm in (A), and 120 μm in (A') and (A").

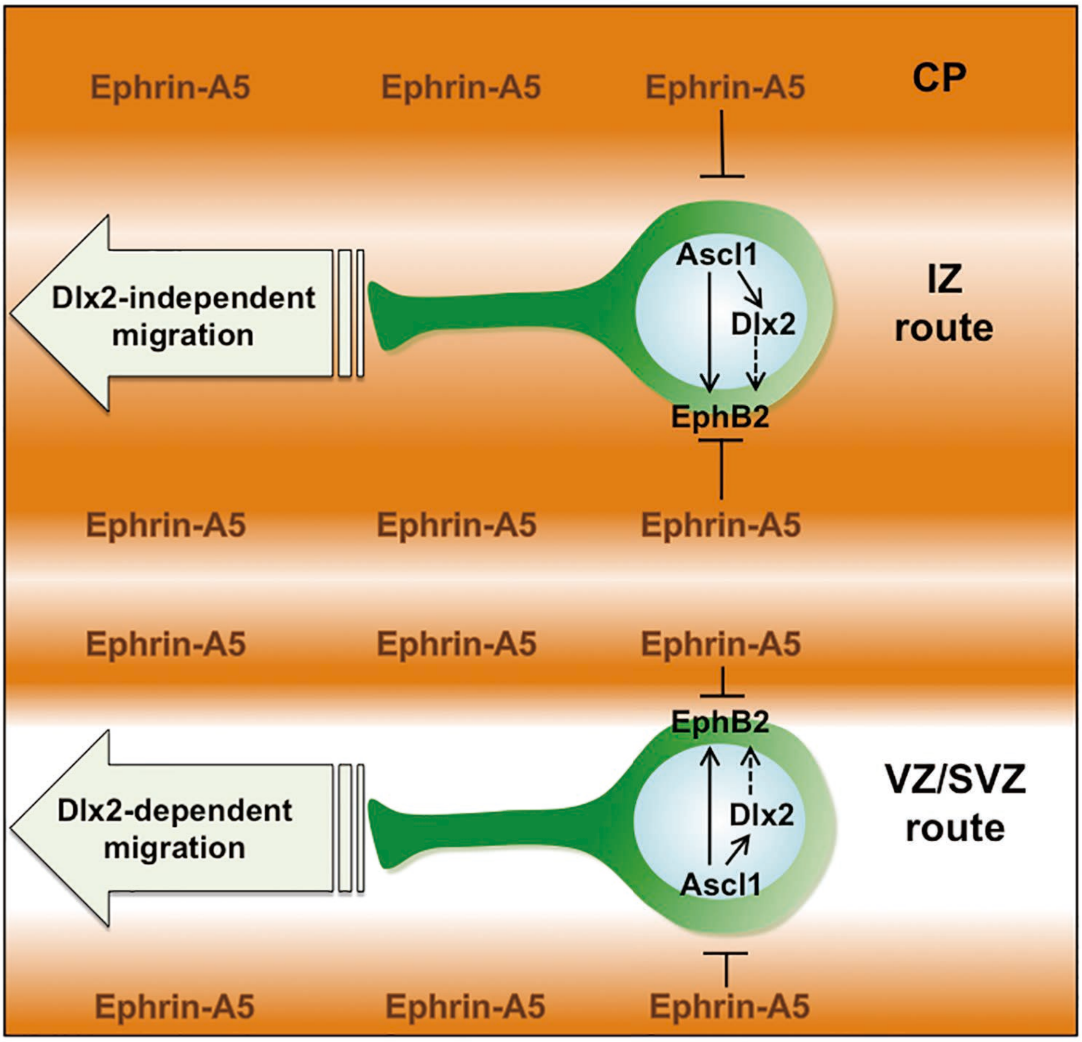


**Figure S12. A schematic model for the role of Ascl1 in the tangential migration of cortical interneurons.** Ascl1 promotes tangential migration through the VZ/SVZ route in a Dlx2-dependent manner, while promoting tangential migration through the IZ route in a Dlx2-independent manner. Ascl1 induces *Ephb2* expression to confine these two migratory routes through EphB2-Ephrin-A5 signaling.

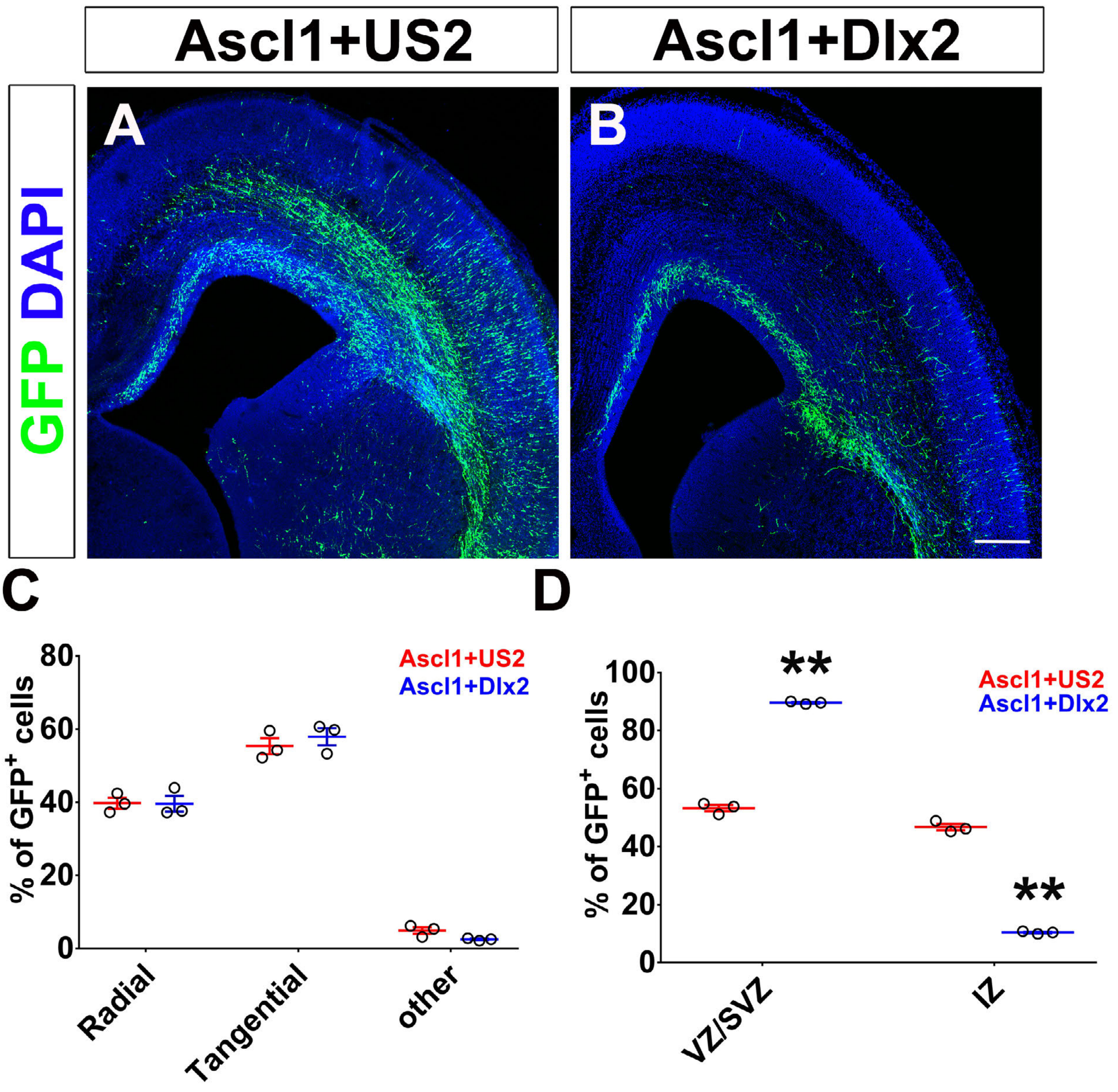


**Figure S13. Simultaneous expression of *Ascl1* and *Dlx2* promotes tangential migration through the VZ/SVZ route.** Four days after electroporation to the dorsal telencephalon, brains of E19.5 rats were dissected, fixed, and sectioned in the coronal plane. Electroporated cells were labeled with anti-GFP in green; nuclear DNA was stained with DAPI in blue. US2 vector was used as a control. (A) In Ascl1+US2 group, many GFP-positive cells were distributed in the VZ/SVZ and IZ dorsomedial to the electroporated site. (B) In Ascl1+Dlx2 group, many GFP-positive cells were distributed in the VZ/SVZ dorsomedial to the electroporated site. Length of the scale bar is 120 μm. (C) Migrating cells were categorized according to the orientation of their leading processes. (D) Quantification of GFP-positive cells in the VZ/SVZ and IZ. Data are presented as mean±SEM with all data points and analyzed by using Student's test, n=3. *: $p<0.05$; **: $p<0.01$.

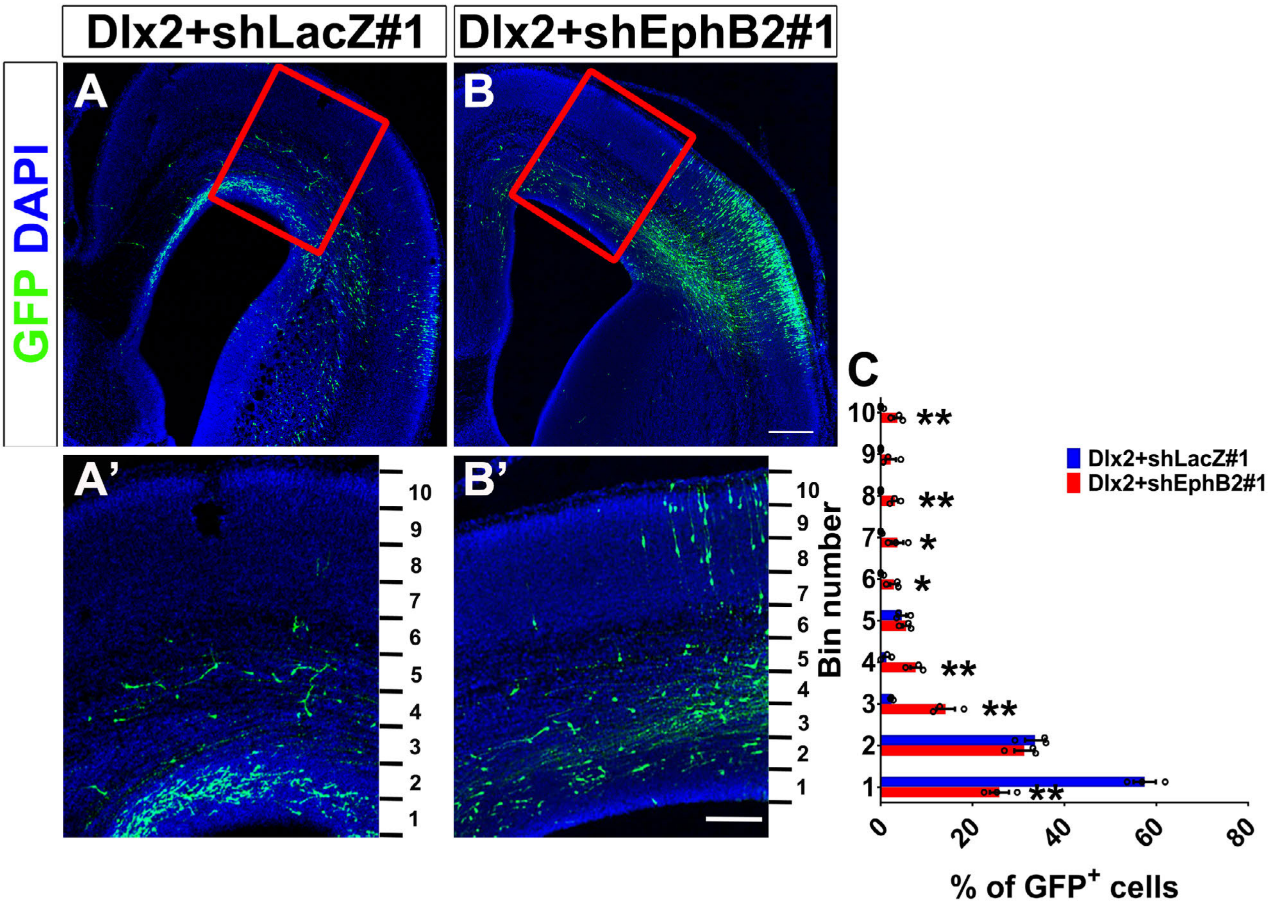


**Figure S14. Knockdown of *Ephb2* disrupts tangential migration promoted by Dlx2.** Four days after electroporation to the dorsal telencephalon, brains of E19.5 rats were dissected and sectioned in the coronal plane. Electroporated cells were labeled with anti-GFP in green; nuclear DNA was stained with DAPI in blue. (A'-B') are zoomed regions of (A-B) indicated by red squares. The cortex was divided equally into 10 bins. (A, A') In Dlx2+shLacZ#1 group, many GFP-positive cells were distributed in the VZ/SVZ (bins 1 and 2). (B, B') In Dlx2+shEphB2#1 group, GFP-positive cells distributed in the VZ/SVZ (bins 1 and 2) were reduced. Length of the scale bar is 120 μm in (A, B) and 100 μm in (A', B'). (C) Distribution of GFP-positive cells in the dorsal telencephalon. Data are presented as mean±SEM with all data points and analyzed by using one-way ANOVA with Tukey's-HSD post hoc test, n=3. *: $p<0.05$; **: $p<0.01$.

**Video S1. GFP-positive cells migrate radially in US2 (Control) group.** A yellow arrowhead indicates a radially migrating cell.

**Video S2. GFP-positive cells migrate tangentially or radially in Ascl1 group.** A red arrow indicates a tangentially migrating cell and a yellow arrowhead indicates a radially migrating cell.
